# TBX3 is essential for establishment of the posterior boundary of anterior genes and upregulation of posterior genes together with HAND2 during the onset of limb bud development

**DOI:** 10.1242/dev.202722

**Published:** 2024-06-03

**Authors:** Geoffrey Soussi, Ausra Girdziusaite, Shalu Jhanwar, Victorio Palacio, Marco Notaro, Rushikesh Sheth, Rolf Zeller, Aimée Zuniga

**Affiliations:** ^1^ Developmental Genetics, Department of Biomedicine, University of Basel, 4058 Basel, Switzerland; ^2^ enGene Statistics GmbH, 4052 Basel, Switzerland

**Keywords:** Enhancer activities, Gene expression boundary, *Gli3*, Irx genes, Limb bud, TBX3 target genes, Mouse

## Abstract

During limb bud formation, axis polarities are established as evidenced by the spatially restricted expression of key regulator genes. In particular, the mutually antagonistic interaction between the GLI3 repressor and HAND2 results in distinct and non-overlapping anterior-distal *Gli3* and posterior *Hand2* expression domains. This is a hallmark of the establishment of antero-posterior limb axis polarity, together with spatially restricted expression of homeodomain and other transcriptional regulators. Here, we show that TBX3 is required for establishment of the posterior expression boundary of anterior genes in mouse limb buds. ChIP-seq and differential gene expression analysis of wild-type and mutant limb buds identifies TBX3-specific and shared TBX3-HAND2 target genes. High sensitivity fluorescent whole-mount *in situ* hybridisation shows that the posterior expression boundaries of anterior genes are positioned by TBX3-mediated repression, which excludes anterior genes such as *Gli3*, *Alx4*, *Hand1* and *Irx3/5* from the posterior limb bud mesenchyme. This exclusion delineates the posterior mesenchymal territory competent to establish the *Shh*-expressing limb bud organiser. In turn, HAND2 is required for *Shh* activation and cooperates with TBX3 to upregulate shared posterior identity target genes in early limb buds.

## Teaser

**Summary:** TBX3 sets the posterior expression boundaries of anterior genes in mouse limb buds by transcriptional repression and promotes posterior identity together with HAND2.

## INTRODUCTION

The developing vertebrate limb bud is an experimental paradigm used to study the fundamental mechanisms, gene regulatory networks (GRNs) and cellular interactions that govern organ development. During limb bud formation, the antero-posterior (AP), proximo-distal (PD) and dorso-ventral (DV) limb bud axes are established, which results in precise positioning of the two main signal centres: the fibroblast growth factor (FGF) signalling apical ectodermal ridge (AER) and the sonic hedgehog (SHH)-signalling posterior mesenchymal organiser (reviewed by Zuniga, 2015). Many of the transcriptional regulators and signalling pathways that control the onset of limb bud development have been identified and functionally analysed (reviewed by Zuniga and Zeller, 2020). One such hallmark is the AP polarisation of the mesenchyme during limb bud formation, which manifests itself by the complementary expression of *Gli3* in the anterior-distal and *Hand2* in the posterior mesenchyme (te Welscher et al., 2002a). These two transcriptional regulators act in a mutually antagonistic manner as genetic inactivation of *Gli3* in mouse limb buds causes anterior expansion of *Hand2* expression and pre-axial (anterior) digit polydactyly (te Welscher et al., 2002a; Lopez-Rios et al., 2012). Conversely, *Gli3* expands posteriorly, and establishment of posterior limb bud identity and *Shh* activation are disrupted in *Hand2-*deficient limb buds, which phenocopies the digit loss observed in *Shh-*deficient mouse limbs (Galli et al., 2010). Initially, the GLI3 repressor isoform (GLI3R; Wang et al., 2000) and HAND2 are co-expressed, but the antagonism results in rapid establishment of their complementary expression domains during limb bud formation (Osterwalder et al., 2014). A study by Lex et al. (2022) showed that the binding of GLI3R to its target *cis*-regulatory modules (CRMs) is inert prior to activation of SHH signalling. In *Gli3*-deficient limb buds at early stages, i.e. when GLI3 repression is removed, enhancer accessibilities, activities and target gene expression are not increased as is the case after the onset of SHH signalling (Lex et al., 2022). These findings contrast with the fact that the mutually antagonistic restriction of *Hand2* and *Gli3* does not require SHH signalling, as both the posterior *Hand2* and anterior *Gli3* restriction occur normally in *Shh*-deficient limb buds (te Welscher et al., 2002a). Furthermore, genetic loss-of-function analysis has shown that first *Hand2* and then SHH signalling are required to overcome the repressive effect of GLI3R the posterior mesenchyme, which is essential for digit patterning and distal progression of limb bud outgrowth (Litingtung et al., 2002; te Welscher et al., 2002b; Zhu et al., 2008; Galli et al., 2010). Therefore, an important open question is whether the *Gli3* and *Hand2* expression boundaries are established by direct cross-regulation between HAND2 and GLI3R or depend on unknown additional transcriptional regulators. The present study addresses posterior boundary formation for anterior-proximal and anterior-distal gene expression during establishment of the AP limb bud axis.

Several transcription factors (TFs) required for activation/upregulation of *Gli3* and *Hand2* have been identified in mouse limb buds. For example, positive regulation of *Gli3* depends on *Irx3/Irx5* and *Sall4*, which impact *Gli3* expression via a specific enhancer active in the anterior mesenchyme (Li et al., 2014; Akiyama et al., 2015). In turn, *Gli3* enhances the expression of *Irx3/Irx5* and *Sall4* as part of a transcriptional feedback mechanism (Yokoyama et al., 2017). The expression of *Hand2* in the posterior limb bud mesenchyme depends on specific homeodomain TFs. Hox9 paralogues are required in a redundant fashion to establish the posterior *Hand2* expression domain in early forelimb buds, whereas *Isl1* acts upstream of *Hand2* in hindlimb buds (Xu and Wellik, 2011; Itou et al., 2012). The PBX1 and PBX2 homeoproteins upregulate *Hand2* expression by interacting with specific enhancers (Capellini et al., 2006; Losa et al., 2023). In the posterior limb bud mesenchyme, PBX1 forms a complex with HAND2 to co-regulate target genes, including several key regulators of mouse limb bud development (Losa et al., 2023). Together, these studies reveal the complexity of gene regulatory interactions among different key players, but fall short of providing insights into the repressive mechanisms that restrict *Gli3* and *Hand2* expression during AP axis polarisation. Osterwalder et al. (2014) identified the HAND2 target CRMs within the genomic landscape of genes functioning during the onset of limb bud development. Moreover, *Tbx3* has been identified as a HAND2 target gene in posterior limb bud mesenchyme, leading to the proposal that, in addition HAND2, *Tbx3* might participate in ‘fine-tuning’ of the posterior *Gli3* expression boundary (Osterwalder et al., 2014), which prompted us to attempt to identify the range of TBX3 target genes in early limb buds (this study).

*Tbx3* is a member of the *Tbx2* sub-family (reviewed by Sheeba and Logan, 2017), expressed first in the lateral plate mesenchyme and subsequently in the posterior and anterior limb bud mesenchyme and AER (Emechebe et al., 2016). Haploinsufficient *TBX3* mutations in humans cause ulnar-mammary syndrome (UMS), which causes a pleiotropic phenotype, including severe reductions of the posterior skeletal elements of the upper extremities (Bamshad et al., 1999). In contrast to human *TBX3* mutations, inactivation of Tbx3 in the mouse causes mid-gestational lethality, which can be circumvented by conditional *Tbx3* inactivation in early mouse limb buds (Davenport et al., 2003; Frank et al., 2013). Similar to the limb skeletal defects observed in humans with UMS, skeletal analysis of *Tbx3*-deficient mouse limb buds shows that posterior digit 5 and the ulna are reduced or lost, but preaxial polydactyly is only detected in mouse mutant limb buds (Bamshad et al., 1999; Emechebe et al., 2016). Molecular analysis shows that *Tbx3* is required in the posterior mesenchyme to upregulate *Hand2* and *Shh* expression (Emechebe et al., 2016), and the subsequent upregulation of posterior *Tbx3* expression becomes dependent on both HAND2 and SHH signalling (Galli et al., 2010). In the posterior limb bud mesenchyme, *Tbx3* is a HAND2 target gene expressed in a more restricted domain than *Hand2* (Osterwalder et al., 2014). In the anterior limb bud mesenchyme, *Tbx3* expression is activated slightly later and depends on SMAD4-mediated BMP signal transduction (Emechebe et al., 2016; Gamart et al., 2021). The anterior TBX3 protein localises to primary cilia and interacts with the SUFU/Kif7 complex to stabilise full-length GLI3 (GLI3FL) and its processing to the GLI3R isoform (Emechebe et al., 2016). In *Tbx3*-deficient limb buds, GLI3 proteins are degraded, which causes a pre-axial polydactyly similar to that of *Gli3*-deficient forelimb buds (Lopez-Rios et al., 2012; Emechebe et al., 2016). The knowledge gained from studying these early gene regulatory interactions in mouse limb buds and associated limb skeletal phenotypes provides important insights into the aetiology of human congenital limb malformations and guides identification of human disease alleles (Tao et al., 2017).

For this study, we have generated a novel mouse *Tbx3* allele with a 3xFLAG epitope tag inserted into the TBX3 protein. This *Tbx3*^3xF^ allele was used for chromatin immunoprecipitation in combination with next generation sequencing (ChIP-seq) in combination with open chromatin analysis (assay for transposase-accessible chromatin with sequencing, ATAC-seq). This, together with differential gene expression analysis (RNA sequencing, RNA-seq) of wild-type and mutant forelimb buds, identified the TBX3 cistrome and target genes during onset of limb bud outgrowth. In parallel, HAND2 target genes were used to pinpoint the target genes shared between TBX3 and HAND2. Spatial analysis of target gene expression and enhancer activities in wild-type and mutant limb buds resulted in several major findings. Fluorescent RNA *in situ* hybridisation revealed that *Tbx3*, but not *Hand2*, is required to establish the precise posterior expression boundaries of anterior genes such as *Gli3*, *Hand1*, *Alx4* and *Irx3/5*. This finding is important in light of previous studies showing that *Hand2* and *Shh* need to override the repressive activity of GLI3R to enable progression of limb bud development (Litingtung et al., 2002; te Welscher et al., 2002b; Galli et al., 2010). Analysis of two CRMs that regulate the spatial *Gli3* expression in mouse limb buds (Osterwalder et al., 2018) has shown that TBX3 directly interacts with and restricts their activity to the posterior *Gli3* expression boundary. This direct interaction excludes *Gli3* transcription from the posterior mesenchymal territory in which *Shh* is activated. Our study identifies an unexpected unique function of TBX3 in positioning AP expression boundaries, which is a crucial step for enabling establishment of the SHH signalling centre. In the posterior mesenchyme, *Tbx3* functions together with *Hand2* in positive regulation of posterior identity and *Shh* pathway genes.

## RESULTS

### Identification of TBX3 target genes in mouse forelimb buds

**Fig. 1.**
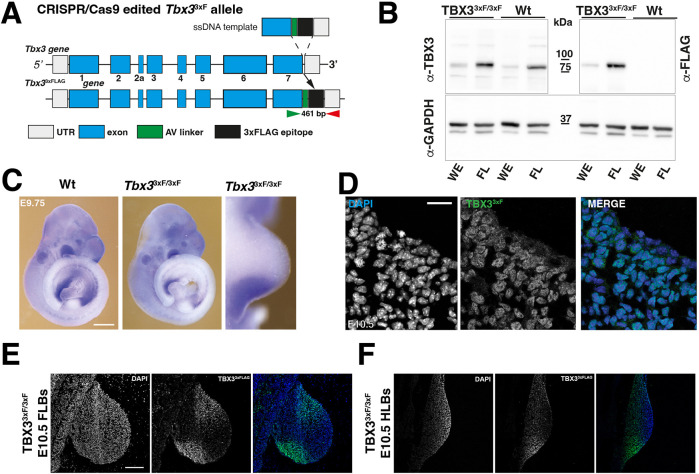
**Generation and characterisation of the *Tbx3*^3xFLAG^ (*Tbx3*^3xF^)-tagged mouse allele.** (A) Scheme summarising the strategy to generate the *Tbx3*^3xFLAG^ allele. Green and red arrowheads indicate the genotyping primers. (B) Western blot analysis of whole embryos (WE) and forelimb bud (FL) protein extracts (E10.5). After transfer, the western blot membrane was cut into three pieces prior to detection of TBX3 proteins and GAPDH as a loading control. Anti-TBX3 antibodies (exposure 30 sec; see Fig. S1B) detect the wild-type (Wt) and 3xFLAG proteins (TBX3^3xF/3xF^; left panel); anti-FLAG antibodies (exposure 4 sec; see Fig. S1A) specifically detect TBX3^3xFLAG^ proteins (right panel, *n*=3). Detection of the GAPDH protein serves as a loading control (exposure 4 sec; see Fig. S1A). Solid lines indicate the cropped areas. For even longer exposure, see Fig. S1C (90 sec). (C) WISH analysis of Wt and *Tbx3*^3xF/3xF^ mouse embryos (*n*=3; 28-30 somites, E9.75). Scale bar: 250 µm. (D-F) Immunofluorescence analysis of limb bud sections (*n*=3; 34-36 somites, E10.5). Scale bars: 20 µm in D; 200 µm in E,F. All forelimb buds are oriented with anterior to the top and posterior to the bottom.

**Fig. 2.**
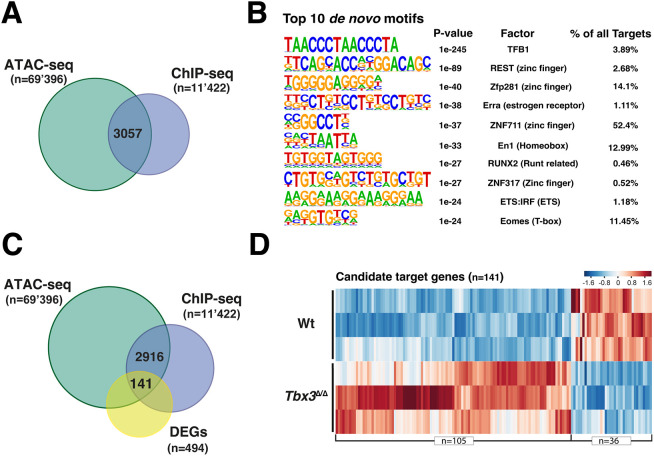
**Identification of TBX3^3xF^ target genes in early mouse forelimb buds.** (A) Overlapping regions of open chromatin (ATAC-seq) and TBX3^3xF^ ChIP-seq peaks identifies 3057 regions encoding potential CRMs. (B) Top 10 *de novo* motif analysis. (C) Intersection of TBX3-bound regions (E9.75-E10.25), ATAC-seq (E9.75) and DEGs (E9.75-E10.0) in wild-type and *Tbx3*^ΔΔ^ forelimb buds identifies 141 TBX3 candidate target genes. (D) Heatmap of the candidate targets genes in wild-type and *Tbx3*^ΔΔ^ forelimb buds. The *z*-score scale represents mean-subtracted regularised log-transformed read counts.

A 3xFLAG (3xF) epitope tag was inserted into the carboxy-terminal part of the mouse *Tbx3* open reading frame. This *Tbx3*^3xF^ allele allows specific detection of endogenous TBX3^3xF^ proteins using FLAG antibodies in mouse wild-type embryos and forelimb buds (Fig. 1A,B, Fig. S1A-C). The insertion of the 3xFLAG epitope tag did not alter the spatial distribution of *Tbx3*^3xF^ transcripts (Fig. 1C) and the TBX3^3xF^ protein was predominantly nuclear in the posterior and anterior limb bud domains [embryonic day (E) 10.5, 33-35 somites; Fig. 1D-F]. *Tbx3*^3xF/3xF^ mice were born at the expected Mendelian ratios and displayed no overt phenotypes. Forelimb buds of *Tbx3*^3xF/3xF^ embryos were used to identify the TBX3 cistrome by ChIP-seq (Fig. 2A, Fig. S1D-F). Two biological replicates consisting each of ∼70 dissected forelimb buds at E9.75-E10.25 (29-32 somites) were analysed. Statistical analysis of the two replicates by model-based analysis of ChIP-seq (MACS) and MSPC identified 11,422 shared TBX3^3xF^ ChIP-seq peaks. Roughly equal fractions of the TBX3^3xF^ ChIP-seq peaks were located within 0-3 kb of the transcriptional start sites (mostly promoter interactions; Zimmerli et al., 2020) and between 3 and 100 kb, which is indicative of intra- and intergenic CRMs with conserved peak summits (Fig. S1D-F, Table S1). Next, the TBX3 peaks located in regions of accessible chromatin were identified by overlapping the ChIP-seq with an ATAC-seq dataset from wild-type forelimb buds at E9.75 (28-29 somites). This identified 3057 TBX3 ChIP-seq peaks located in open chromatin, which is a hallmark of promoters and CRMs (Fig. 2A, Table S2). HOMER *de novo* and known motif enrichment analysis (Fig. 2B, Fig. S2A) identified a variety of enriched TF-binding motifs, including the *Eomes* T-box binding motif (Fig. 2B). The core region of the *Eomes* motif is very similar to if not identical to other Tbx motifs, including the *Tbx3* motif (Fig. S2B). Furthermore, the genomic regions enriched in TBX3 chromatin complexes also encode binding sites for other TFs, such as zinc finger and homeodomain proteins, which points to possible co-regulation (Fig. 2B).

Differentially expressed genes (DEGs) between wild-type and *Tbx3*-deficient mouse forelimb buds (homozygous for the *Tbx3*^ΔVenus^ allele; Kunasegaran et al., 2014) were identified as follows (Fig. S2C). RNA-seq analysis of three independent biological replicates of forelimb bud pairs from wild-type and *Tbx3*^ΔΔ^ embryos (E9.75-10.0, 28-31 somites) identified a common set of 494 DEGs with a fold change >1.2 (*P*≤0.05; Table S3). Among the 494 DEGs, 357 were up- and 137 downregulated in *Tbx3*^Δ/Δ^ mouse forelimb buds (Fig. S2C). The TBX3 target genes were identified by mapping the ChIP-seq peaks located within accessible chromatin to the nearest gene(s) located within ≤1 MB interval using GREAT analysis (Fig. 2C,D). Among these, differential expression identified 141 TBX3-dependent candidate target genes in early mouse forelimb buds at E9.75-E10.0 (Fig. 2D, Table S4). The majority of the TBX3 candidate targets genes were upregulated (105 of 141; Fig. 2D), and the remainder were downregulated in mutant limb buds (36 of 141; Fig. 2D). Gene Ontology (GO) analysis revealed the predominance of genes functioning in embryonic and limb bud development (Fig. S2D,E). Forty-one of the 141 TBX3 target genes (28%) were genes co-regulated by SHH signalling during subsequent limb bud outgrowth (E10.5; Table S6; Probst et al., 2011). Together, these results indicate that TBX3 functions mostly, but not exclusively, in restricting/repressing gene expression either directly (Fig. 2D) or indirectly (Fig. S2C), likely as part of the GRNs that control early forelimb bud development (reviewed by Zuniga and Zeller, 2020).

### *Tbx3* regulates the spatial expression domains of diverse transcriptional regulators with key functions during early limb bud development

**Fig. 3.**
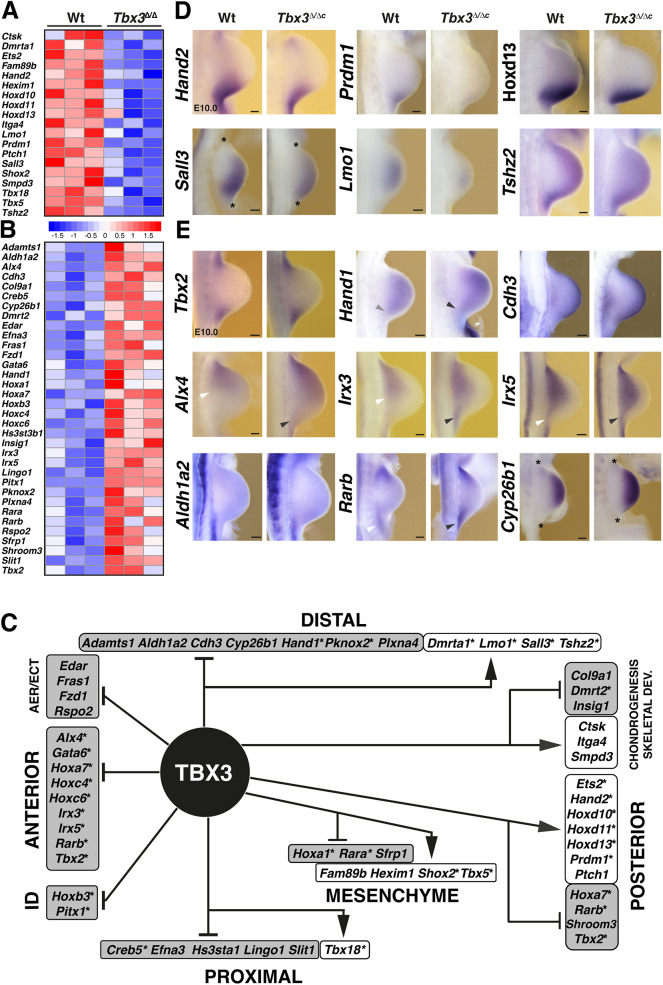
**TBX3 candidate target gene regulatory network in early limb development.** (A,B) Heatmaps illustrating relative gene expression of developmental regulators in wild-type (Wt) and *Tbx3*-deficient samples (*n*=3 biological replicates, E9.75-E10.0). The *z*-score scale represents mean-subtracted regularised log-transformed read counts. Shown are genes (*n*=53) that have essential functions in limb buds (Table S5). (C) TBX3 target GRN for early limb buds. In addition, target genes expressed in the AER and ectoderm (ECT), functioning in limb identity (ID) or chondrogenesis and skeletal development are shown. Arrows point to genes for which expression is positively regulated by TBX3 (white boxes), and inhibitory lines indicate genes for which expression is repressed (grey boxes). Asterisks indicate TFs. (D,E) WISH analysis of select genes in wild-type and *Tbx3*^Δ/Δc^ mouse forelimb buds (E9.75-E10.25, 29-32 somites). Asterisks indicate limb bud margins for some limb buds (D). Arrowheads indicate the posterior expression boundaries (*Alx4*, *Irx3*, *Irx5*) or upregulation (*Hand1*) in wild-type (white) and mutant (grey) limb buds. Forelimb buds are oriented with anterior to the top and posterior to the bottom. Scale bars: 100 µm.

Next, the fraction of TBX3 candidate target genes with known spatial expression patterns and/or essential functions during limb development were manually curated (Fig. 3A,B, Table S5). This analysis identified 19 genes for which expression was positively regulated (Fig. 3A), and 34 genes repressed by TBX3 in wild-type limb buds (Fig. 3B). This gene annotation analysis (Table S5) allowed construction of a TBX3-target GRN for early limb buds (Fig. 3C). At these stages, *Tbx3* is expressed predominantly in the posterior mesenchyme, with little to no expression in the anterior mesenchyme (E9.75-E10.25; Emechebe et al., 2016; see also Fig. 6A). The GRN analysis showed that the majority of target genes expressed in the anterior and proximal mesenchyme and AER are repressed by TBX3. This was also the case for several genes expressed in the distal mesenchyme, whereas most TBX3 target genes expressed in the posterior mesenchyme are positively regulated. Together, these analyses indicate that posterior TBX3 largely governs the target GRN in early limb buds, although a potential contribution from anteriorly expressed TBX3 cannot be ruled out. Moreover, the majority of TBX3 target genes are TFs (30 of 43 genes; indicated by asterisks in Fig. 3C), which significantly expands the TF interactions governing onset limb bud outgrowth (reviewed by Zuniga and Zeller, 2020).

To assess spatial changes in target gene expression, *Tbx3* was inactivated in the mouse limb bud mesenchyme using the *Prrx1*-CRE driver (Logan et al., 2002) in mouse embryos carrying the *Tbx3*^ΔVenus^ allele and a conditional *Tbx3*^flox^ allele (Fig. S3A; Frank et al., 2012). Whole-mount immunofluorescence analysis of *Tbx3*^Δ/Δc^ forelimb buds showed that the TBX3 protein is cleared from limb bud mesenchyme by E10.0, but it remains in the AER (Fig. S3B). Of note, fluorescent whole-mount *in situ* hybridisation chain reaction (HCR™) analysis (RNA-FISH) detected variable levels of the non-functional *Tbx3*^ΔVenus^ transcripts in *Tbx3*^Δ/Δc^ forelimb buds (Fig. S3B, E10.5). This conditional inactivation caused pre-axial duplications of digit 1 in combination with hypoplasia or loss of posterior digit 5 and variable defects of proximal limb skeletal elements (ulna, radius and humerus; Fig. S3C; Emechebe et al., 2016).

Whole-mount RNA *in situ* (WISH) analysis of specific mesenchymal TBX3 targets revealed that not only transcript levels (Fig. 3A,B) but also the spatial expression boundaries of several genes are altered in *Tbx3*^Δ/Δc^ forelimb buds at E10.0 (Fig. 3D,E). For example, the reduced expression of several posterior target genes (Fig. 3A) was paralleled by more posteriorly (*Hand2*, *Prdm1*, *Hoxd11*, *Hoxd13*, *Ets2*) or distally (*Sall3*, *Lmo1*) restricted domains, whereas *Tshz2* expression was more diffuse in *Tbx3*^Δ/Δc^ than in wild-type forelimb buds (Fig. 3D; see Fig. 5C for *Ets2*). The expression domains of all these target genes overlapped at least partially with the posterior *Tbx3* domain (Fig. 1C), but it is likely that downstream effects as part of the TBX3-target GRN contribute to the overall reduction of *Sall3* and *Lmo1* expression in the distal mesenchyme (Fig. 3C,D). Next, we analysed the spatial expression of target genes upregulated in *Tbx3*-deficient forelimb buds (Fig. 3B,E). Although detection of transcriptional upregulation by WISH is more challenging than downregulation (Gamart et al., 2021), spatial changes were observed for several target genes in *Tbx3*^Δ/Δc^ forelimb buds (Fig. 3B,E). In particular, the expression of several transcriptional regulators expressed at the highest levels in the anterior mesenchyme, namely *Hand1*, *Alx4*, *Irx3* and *Irx5*, was posteriorly expanded in mutant forelimb buds (Fig. 3E, arrowheads; Fig. S4A). Functional annotation of target genes showed that TBX3 negatively regulates key components of the retinoic acid (RA) pathway in early limb buds, namely the RA synthesising enzyme *Aldh1a2* (*Raldh2*), the receptors *Rara* and *Rarb* and the RA-degrading enzyme *Cyp26b1*, as they are upregulated in *Tbx3*^Δ/Δc^ forelimb buds (Fig. 3B, Table S5). However, only two of these genes, *Rarb* and *Cyb261b*, were expressed in the mesenchyme of wild-type forelimb buds, whereas *Aldh1a2* is expressed in the posterior flank mesenchyme but did not expand into the mesenchyme of *Tbx3*^Δ/Δc^ forelimb buds (Fig. 3E, lower panel). *Rarb* was upregulated and its expression boundary changed in the posterior-proximal mesenchyme (Fig. 3E, arrowheads). Concurrently, the *Cyp26b1* expression was less distally restricted, which indicates that RA pathway activity could be altered. As previous analysis has shown that AER-FGFs upregulate *Cyp26b1* expression (Probst et al., 2011), we assessed *Fgf8* and *Fgf4* AER expression in wild-type and *Tbx3*^Δ/Δc^ forelimb buds, but no changes were detected (Fig. S4B). Taken together, these results show that TBX3 preferentially downregulates/represses target genes in the anterior and distal mesenchyme, whereas most posterior target genes are positively regulated (Fig. 3C). The posterior expansion of *Hand1*, *Alx4* and *Irx3/5* and the enlargement of the *Rarb* domain (Fig. 3E, arrowheads) points to a TBX3 repressor function in gene expression boundary formation in the posterior limb bud mesenchyme.

### Shared target genes reveal distinct roles for *Tbx3* and *Hand2* in regulating posterior identity

**Fig. 4.**
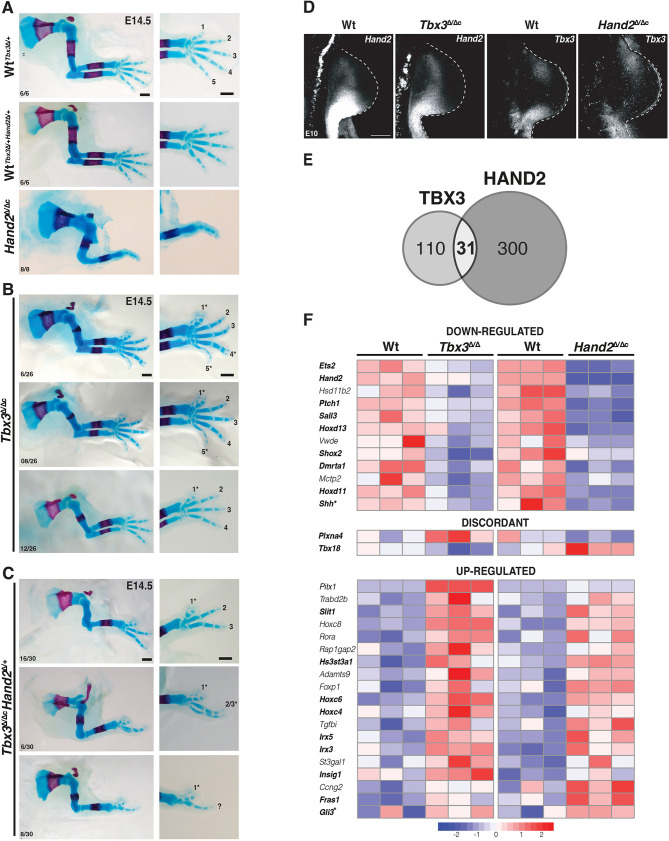
***Tbx3* and *Hand2* cooperatively control posterior limb skeletal identities and co-regulate shared target genes in early limb buds.** (A-C) Skeletal analysis of wild-type (Wt), *Hand2*-deficient (A), *Tbx3*^Δ/Δc^ (B) and *Tbx3*^Δ/Δc^*Hand2*^Δ/+^ (C) mouse forelimbs (E14.5). The genetic interaction of *Tbx3* and *Hand2* regulates posterior limb skeletal elements (ulna and posterior digits; C). The number of embryos exhibiting the illustrated phenotype is indicated at the bottom of each panel. Digit identities are indicated from anterior to posterior (1-5). Asterisks indicate digit malformations. Scale bars: 500 µm. (D) RNA-FISH analysis of forelimb buds (*n*=4 biological replicates per genotype; E10.0; 29-32 somites). All limb buds are oriented with anterior to the top and posterior to the bottom. Scale bar: 200 µm. (E) TBX3 and HAND2 share a small subset of their target genes (*n*=31). (F) Heatmap of the target genes co-regulated by TBX3 and HAND2. Genes in bold have known functions in early limb buds. Asterisks indicate that *Shh* and *Gli3* are manually curated target genes.

Previous analysis showed that *Tbx3* and *Hand2* participate in restricting *Gli3* expression from the posterior mesenchyme, but did not resolve the genetic and molecular hierarchies and the general extent to which these two TFs impact spatial gene expression during early forelimb bud development. In fact, *Tbx3* and *Hand2* upregulate each other's expression in the posterior limb bud mesenchyme, which is indicative of feed-forward regulation (Fig. 3A,D). To gain insight into the functional relevance of the *Tbx3*-*Hand2* interactions, the skeletal phenotypes of mouse embryos lacking *Hand2*^Δ/Δc^*Tbx3*^Δ/Δc^ and *Tbx3*^Δ/Δc^*Hand2*^Δ/+^ in forelimb buds were comparatively analysed at E14.5 (Fig. 4A-C). *Hand2*^Δ/Δc^ forelimb buds displayed characteristic distal limb skeletal truncations and absence of the ulna (Fig. 4A) and *Tbx3* expression was lost from the posterior limb bud mesenchyme by E10.0 (Fig. 4D; Osterwalder et al., 2014). In contrast, *Tbx3*^Δ/Δc^ forelimbs exhibited duplications of the distal phalanges and metacarpals of digit 1, together with digit 5 hypoplasia or agenesis (Fig. 4B, digit phenotypes indicated by asterisks; Emechebe et al., 2016). In addition, variable degrees of deltoid tuberosity agenesis, ulnar hypoplasia and thickening of the radius were detected (Fig. 4B; Lopatka and Moon, 2022). *Hand2* remained expressed at lower levels in the posterior mesenchyme of *Tbx3*^Δ/Δc^ forelimb buds (Fig. 4D,F). Inactivation of one *Hand2* allele in *Tbx3*-deficient limbs caused more severe skeletal defects, including loss of metacarpals and digits (Fig. 4C). The most severe phenotypes had striking similarities with the *Hand2* loss-of function phenotypes, with the exception of the digit 1 duplications, which are a hallmark of *Tbx3*-deficient forelimbs (Fig. 4C, bottom panel, compare with Fig. 4B). This genetic analysis shows that *Hand2* and *Tbx3* interact in the posterior limb bud mesenchyme, but that the *Tbx3-*specific functions in the anterior mesenchyme are not dependent on *Hand2* (Fig. 4D). As *Tbx3* expression is lost from the posterior mesenchyme of *Hand2*-deficient limb buds, the comparative analysis of *Tbx3*^Δ/Δc^ and *Hand2*^Δ/Δc^ forelimb buds should pinpoint *Tbx3*-specific and shared *Tbx3*-*Hand2* functions in regulation of shared target genes.

The HAND2 target genes in early limb bud were identified by re-analysing the ChIP-seq dataset by Osterwalder et al. (2014) in combination with RNA-seq analysis as follows. Only HAND2 ChIP-seq peaks located in open chromatin regions (ATAC-seq) were analysed in combination with genes expressed differentially in wild-type and *Hand2* ^Δ/Δc^ forelimb buds (E10.0, *n*=3 biological replicates per genotype; Table S7). This analysis identified 331 differentially expressed HAND2 candidate target genes with a fold change ≥1.2 (*P*≤0.05; Fig. S5, Table S8). In *Hand2* ^Δ/Δc^ forelimb buds, 124 target genes were down- and 209 upregulated (Fig. S5). The common TBX3 and HAND2 target genes were identified by overlapping the *Tbx3* and *Hand2* datasets, which identified 31 target genes that are differentially expressed in both *Tbx3-* and *Hand2*-deficient limb buds (E10.0; Fig. 4E,F, Table S9). Two additional genes, *Shh* and *Gli3*, the expression of which is known to depend critically on TBX3 and HAND2, were added to the list of shared target genes by manual curation (Fig. 4F, asterisks, Table S9) for the following reasons: *Shh* has been previously identified as a HAND2 target gene (Galli et al., 2010) and its expression is significantly downregulated in both *Tbx3* and *Hand2* mutant limb buds (Table S9). However, it is notoriously difficult to detect interactions with the distant *Shh* limb bud enhancer ZRS (≥800 Mb; Lettice and Hill, 2005) by ChIP-seq analysis for HAND2 (Osterwalder et al., 2014) and TBX3 as a ChIP-seq peak is only called in one replicate. The latter is due to *Shh* being expressed by only a small fraction of mesenchymal cells in early limb buds. *Gli3* is a HAND2 target gene (Osterwalder et al., 2014) and this study establishes that TBX3 interacts with several *Gli3* enhancers (see below). Nevertheless, *Gli3* was not scored as a DEG as its upregulation fell just below the threshold (>1.2), with a 1.15-fold upregulation in *Tbx3*^Δ/Δc^ and 1.2-fold increase in *Hand2*^Δ/Δc^ forelimb buds (Table S9).

Of all candidate target genes, two were regulated in a discordant manner by TBX3 and HAND2 (Fig. 4F, middle panel). All other targets were concordantly controlled by both TFs with their expression being either enhanced or reduced in early forelimb buds. Nine of the 12 target genes downregulated in *Tbx3*^Δ/Δc^ and *Hand2*^Δ/Δc^ forelimb buds were expressed in the posterior and/or distal mesenchyme (Fig. 4F, bold, top panel). Furthermore, nine of 19 target genes upregulated in both mutants have distinct spatial expression patterns and/or essential functions in early limb buds (Fig. 4F, bold, bottom panel). Seven of these are either expressed in the anterior/anterior-distal (*Hoxc4*, *Hoxc6*, *Irx3*, *Irx5*, *Gli3*) or proximal limb bud mesenchyme (*Hs3st3a1* and *Slit1*). In addition, the interactions of TBX3 and HAND2 with CRMs associated with the shared target genes were mapped (Fig. S6). This established that most target genes (∼70%) are regulated by CRMs that either interact with HAND2 or TBX3. Only ∼30% of all target gene loci included at least one CRM that is enriched in both HAND2 and TBX3 chromatin complexes (Fig. S6). Taken together, this analysis shows that *Hand2* and *Tbx3* co-regulate major TFs in the GRN that orchestrates limb axes patterning and restricts *Shh* expression to the posterior mesenchyme.

**Fig. 5.**
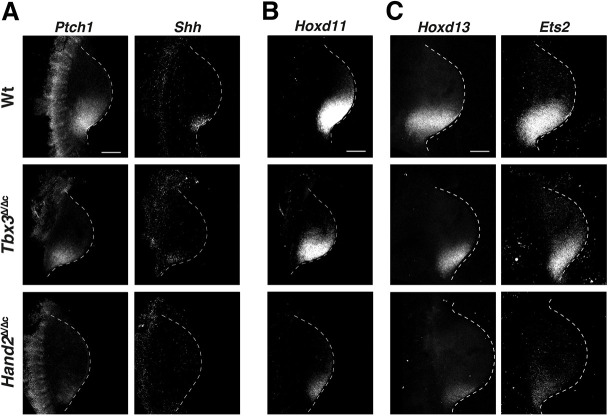
***Hand2* and *Tbx3* requirement for activation and upregulation of key regulators involved in establishment of posterior identities.** (A) RNA-FISH analysis of the shared targets *Ptch1* and *Shh* in wild-type (Wt), *Tbx3*^Δ/Δc^ and *Hand2*^Δ/Δc^ forelimb buds (E10.0; 29-32 somites). (B,C) RNA-FISH analysis of *Hoxd11* (B), *Hoxd13* and *Ets2* (C) in wild-type and mutant forelimb buds. *Hoxd13* and *Ets2* were colocalised; *Hoxd11* was analysed using different limb buds. All forelimb buds are oriented with anterior to the top and posterior to the bottom. For all genes, *n*=4 biological replicates per genotype were analysed. Dashed lines delineate the shape of the limb bud. Scale bars: 200 µm.

Next, the specific and shared effects of HAND2 and TBX3 on spatial expression of target genes was assessed by RNA-FISH, which allows for precise colocalisation of gene expression. We found that TBX3 and HAND2 directly impact the spatial expression of *Shh* and its receptor *Ptch1* (Fig. 5A). In *Tbx3*^Δ/Δc^ forelimb buds, both *Shh* and *Ptch1* were downregulated and their domains were more posteriorly restricted (Fig. 5A, top and middle panels). In *Hand2*^Δ/Δc^ forelimb buds, *Shh* and *Ptch1* expression were reduced even more (Fig. 5A, lower panels; Galli et al., 2010). As HAND2 and TBX3 are required for activation and upregulation of *Shh* expression, respectively, the concurrent loss of *Ptch1* is indicative of disruption of SHH signalling in *Hand2*^Δ/Δc^ forelimb buds. In addition to HAND2, posterior 5′ Hox genes, including *Hoxd11* and *Hoxd13*, function in *Shh* activation (Kmita et al., 2005), whereas *Ets2* acts downstream of *Hand2* and *Tbx3* to upregulate *Shh* expression (Lettice et al., 2012). In *Tbx3*^Δ/Δc^ forelimb buds, the spatial domains of *Hoxd11/13* and *Ets2* were only slightly more restricted (Fig. 5B,C, top and middle panels), but they were reduced to a small domain in *Hand2*^Δ/Δc^ forelimb buds (Fig. 5B,C, lower panels). The drastic reduction of posterior Hoxd gene expression points to feedback regulation between these genes and *Hand2*. Together, these data indicate that HAND2 functions in activation/initial upregulation of posterior target genes, whereas TBX3 participates in enhancing expression.

**Fig. 6.**
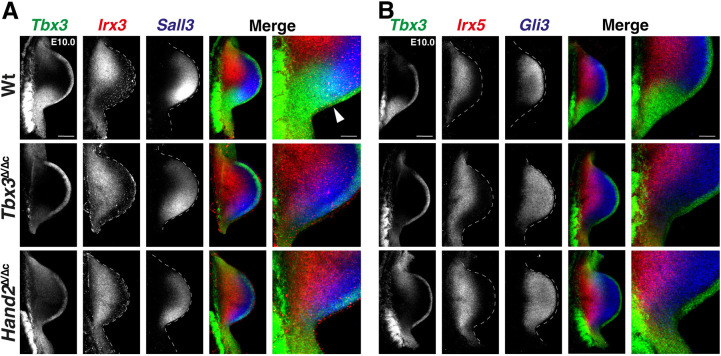
**The essential function of TBX3 in posterior expression boundary formation is independent of HAND2.** (A) Colocalisation of *Tbx3* (green), *Irx3* (red) and *Sall3* (blue) in wild-type (Wt), *Tbx3*^Δ/Δc^ and *Hand2*^Δ/Δc^ forelimb buds (E10.0, 29-32 somites). *n*=4 biological replicates were analysed per gene and genotype. White arrowhead points to the region of overlap between *Tbx3* (green) and *Sall3* (blue) expression. (B) Colocalisation of *Tbx3* (green), *Irx5* (red) and *Gli3* (blue) expression in forelimb buds (E10.0, 29-32 somites). *n*=3 biological replicates were analysed per gene and genotype. Right-most panels in A and B show enlargements of the posterior-proximal regions. All limb buds are oriented with anterior to the top and posterior to the bottom. Dashed lines delineate the shape of the limb bud. Scale bars: 200 µm (main panels); 67 µm (enlargements).

Next, potential changes in the spatial expression of shared target transcript factors in the distal and anterior/proximal limb bud mesenchyme was assessed. In particular, RNA-FISH showed that posterior *Tbx3* and anterior-proximal *Irx3* are expressed in complementary domains in early forelimb buds, whereas the *Tbx3* and anterior-distal *Sall3* expression domains overlap in their posterior boundary region (E10.0; Fig. 6A, top panels). In *Tbx3*^Δ/Δc^ forelimb buds, the spatial domain of *Irx3* was expanded to the posterior limb bud margin (Fig. 6A, *Irx3*, middle panel), which contrasts with the lack of posterior expansion of the *Sall3* domain (Fig. 6A, *Sall3*, middle panel). This is interesting as there is no distinct posterior boundary between the *Tbx3* and *Sall3* expression domains in wild-type forelimb buds (Fig. 6A, top panels). In addition, the PD boundaries between the *Irx3* and *Sall3* expression domains were maintained (Fig. 6A, middle panel). In *Hand2*^Δ/Δc^ forelimb buds lacking posterior *Tbx3* expression (Fig. 4D), no additional alterations in the spatial expression of *Irx3* and *Sall3* were detected (Fig. 6A, bottom panel, compare with middle panel). These results indicate that the posterior expression boundary of *Irx3* (but not *Sall3*) is regulated by TBX3-mediated transcriptional repression. To substantiate this assumption, we analysed the anterior-proximal expression domain of *Irx5* and the anterior-distal expression domain of *Gli3* (Fig. 6B), which were also upregulated in mutant forelimb buds (Fig. 4F). Their spatial expression boundaries were complementary and non-overlapping with *Tbx3* in the posterior mesenchyme (Fig. 6B, top panels). Both *Irx5* and *Gli3* expression expanded to the posterior margin in *Tbx3*^Δ/Δc^ and *Hand2*^Δ/Δc^ forelimb buds, but their PD complementarity was maintained (Fig. 6B, middle and bottom panels; see Fig. S7 for *Gli3*). These results uncover a specific requirement for TBX3 in posterior gene expression boundary formation of anterior genes during AP limb axis polarisation. The results shown in Figs 5 and 6 establish that TBX3-dependent boundary formation excludes both anterior-proximal and anterior-distal genes from the posterior mesenchyme in which the SHH signalling limb organiser is established. Together, these analyses reveal (1) the crucial repressive function of TBX3 in establishing the posterior expression boundaries of several anteriorly expressed transcriptional regulators (Figs 4E and 6) and (2) that TBX3 participates in transcriptional upregulation of posterior identity genes together with HAND2 (Fig. 5).

### TBX3 controls posterior gene expression boundary formation by direct repression of enhancer activities

The AP graded *Gli3* expression in mouse limb buds (Fig. 6B) is controlled by two functionally redundant enhancers embedded in the large *Gli3* genomic landscape (Osterwalder et al., 2018). This provides a unique opportunity to determine the extent to which TBX3 repression might directly impact the spatial activities of these two enhancers. Initially, the spatial activities of three *Gli3* CRMs encoding genomic regions enriched in both TBX3 and HAND2 (VISTA mouse enhancer *mm1179*) or specifically TBX3 chromatin complexes (VISTA mouse orthologue enhancer *mm-hs1586* and *mm652*) were assessed by mouse embryonic *lacZ* reporter assays (Fig. S8; Visel et al., 2007). Activity of the *mm652* enhancer was detected transiently in a fraction of transgenic founder embryos during early hindlimb bud development (E10.5; Fig. S8A, right-most panel) and was not analysed further. In contrast, the functionally redundant *mm1179* and *mm-hs1586* enhancers possessed robust activity in forelimb buds (Fig. S8B, upper panels; Osterwalder et al., 2018). Therefore, the most conserved bases in the predicted TBX3 and HAND2 binding sites were mutated to disrupt TF binding (Figs S8C and S9). *lacZ* reporter analysis showed that the activity of both mutant enhancers is more restricted but fails to expand posteriorly (Fig. S8B). This indicates that, in mouse limb buds, these two *Gli3* enhancers depend on a combination of both positive and repressive trans-regulatory inputs from different T-box family TFs. The repressive effects on the *hs-mm1586* limb bud enhancer were apparent from ectopic activity of the mutant enhancer in eye primordia and facial tissues co-expressing *Tbx3* and *Gli3* (Fig. S8D).

**Fig. 7.**
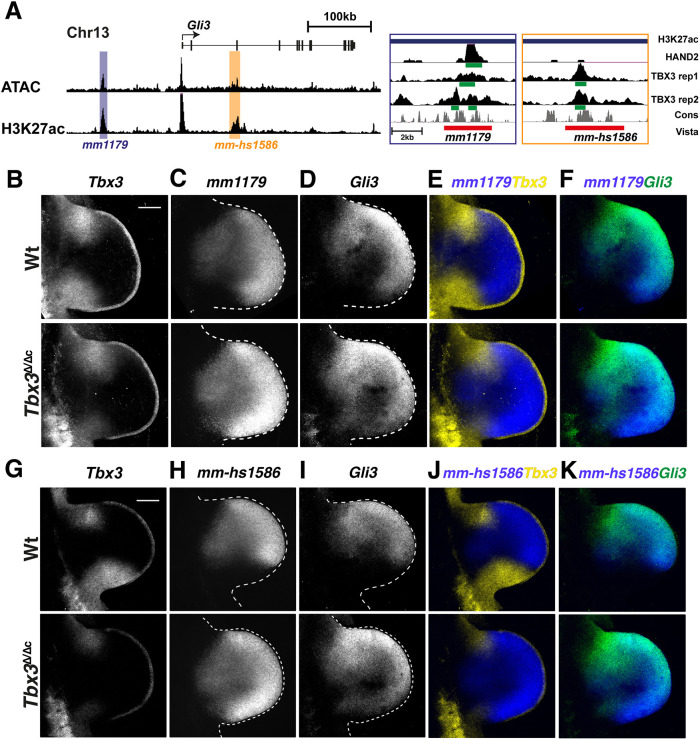
***Tbx3* is required to restrict the activity of the *Gli3* limb enhancers and *Gli3* expression from the posterior limb bud mesenchyme.** (A) UCSC browser view of parts of the *Gli3* genomic landscape with regions of accessible and active chromatin tracks (ATAC-seq and H3K27ac peaks; Malkmus et al., 2021). The two limb enhancers *mm1179* and *mm-hs1586* are highlighted. The enlargements on the right show the genomic regions tested for *Gli3* enhancer activity (red bar) and the HAND2 and TBX3 ChIP-seq peaks. The green bars indicate the called peaks. Both replicates for the TBX3 ChIP-seq are shown; the HAND2 ChIP-seq data is from Osterwalder et al. (2014). (B-K) RNA-FISH analysis of wild-type (Wt) and *Tbx3*^Δ/Δc^ mouse limb buds (E10.5, 35-37 somites) of endogenous *Tbx3* (yellow in E,J), transgenic enhancer-*lacZ* reporter RNA expression (blue in E,F,J,K; *mm1179* in C,E,F and *mm-hs1586* in H,J,K) and endogenous *Gli3* expression (green in F,K). *n*=5 biological replicates were analysed per probe and genotype. Note: low and variable levels of remaining non-functional *Tbx3* ^Δ^ transcripts are detected in *Tbx3*^Δ/Δc^ limb buds by the HCR^TM^ probe set. All forelimb buds are oriented with anterior to the top and posterior to the bottom. Dashed lines delineate the shape of the limb bud. Scale bars: 200 µm.

As this approach was not informative with respect to specific TBX3 functions in posterior boundary formation in limb buds, we generated stable transgenic reporter mouse strains for the *mm1179* and *mm-hs1586* enhancers (Fig. 7A) that reproduce their spatial activities. These *lacZ* reporter transgenes were crossed into *Tbx3*^Δ/Δc^ limb buds and analysed in comparison with wild-type controls by RNA-FISH (Fig. 7B-K). The activities of both enhancers were restricted to the posterior boundary set by the *Tbx3* expression domain in wild-type limb buds, but their activities expanded to the posterior margin in *Tbx3*-deficient forelimb buds (*mm1179*: compare Fig. 7B,C,E; *mm-hs1586*: Fig. 7G,H,J; Movies 1, 2). The posterior expansion of enhancer activity perfectly matched the expanded *Gli3* expression in *Tbx3*^Δ/Δc^ forelimb buds (*mm1179*: compare Fig. 7C,D,F; *mm-hs1586*: Fig. 7H,I,K). This, together with the TBX3 ChIP-seq peaks in both enhancers (Fig. 7A), shows that TBX3 binds these enhancers to repress *Gli3* from the posterior mesenchyme. In *Tbx3*-deficient forelimb buds, the enhancer activity was expanded and increased in comparison with wild-type limb buds (Fig. 7C,H), which underscores the strong repressive effect of TBX3. In summary, this analysis reveals that TBX3 controls the posterior *Gli3* expression boundary and several additional TFs, such as *Alx4*, *Hand1*, *Rarb* and *Irx3/5*, by repressing them from the posterior-proximal limb bud mesenchyme (Figs 3E, 6, Fig. S7).

## DISCUSSION

**Fig. 8.**
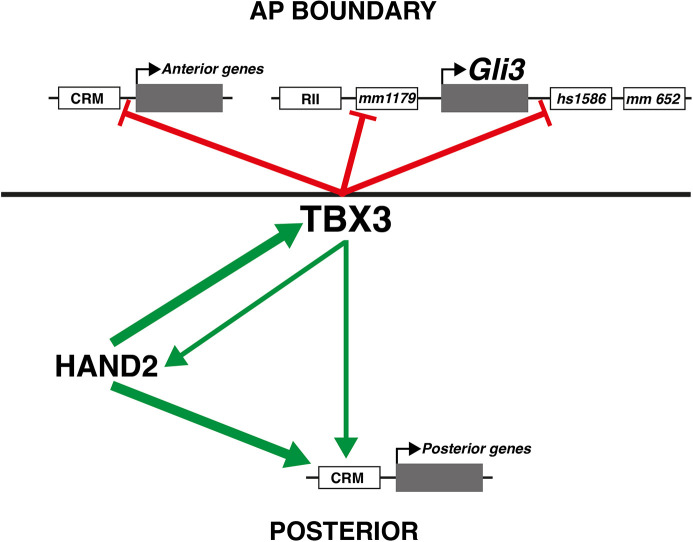
**TBX3 restricts the posterior boundary of anteriorly expressed genes and TBX3 interacts with HAND2 to upregulate posterior gene expression.** Scheme summarising the major findings of this study. TBX3 functions in AP boundary formation by restricting anterior genes from being expressed in the posterior limb bud mesenchyme (red lines). The four limb bud enhancers for *Gli3* are indicated (*mm1179* and *mm-hs1586*, Fig. 7A; *mm682*, Fig. S8; RII: Bastida et al., 2020). Two of these are directly repressed by TBX3 (*mm1179* and *mm-hs1586*). In the posterior limb bud mesenchyme, TBX3 interacts with HAND2 to upregulate the expression of key regulatory genes (green arrows). HAND2 is required for posterior *Tbx3* expression, and TBX3 in turn reinforces posterior *Hand2* expression. The predominant role of HAND2 in positive regulation of posterior genes is indicated by thicker green arrows. All transcription units are shown in grey; CRMs and enhancers are indicated by white boxes.

Transcriptional repression of *Gli3* is a hallmark of the establishment of the posterior mesenchymal territory competent to activate *Shh* expression (Litingtung et al., 2002; te Welscher et al., 2002b). This requires the interaction of HAND2 and several homeodomain transcriptional regulators (5′ HOXD and PBX) with the distant ZRS *Shh* enhancer (Capellini et al., 2006; Galli et al., 2010; Losa et al., 2023). To gain insight into the gene regulatory logic underlying TBX3 functions, we identified its direct transcriptional targets in the mesenchyme of early forelimb buds. The fraction of target genes upregulated in *Tbx3*-deficient limb buds (75%) reveals its predominant role as a transcriptional repressor. It is known that TBX3 interacts with T-box motifs and represses transcription by interaction of its carboxy-terminal domain with HDAC histone deacetylases (Yarosh et al., 2008). In early forelimb buds, *Tbx3* expression in the posterior mesenchyme precedes its anterior activation (Emechebe et al., 2016; this study). The TBX3 target GRN reveals two distinct features of gene regulation at early stages (Fig. 8): (1) the expression of several anterior TBX3 target genes is restricted from the posterior limb bud mesenchyme, revealing the TBX3 requirement for posterior expression boundary formation (Fig. 8, red lines indicating repression); and (2) a significant fraction of genes upregulated by TBX3 in the posterior mesenchyme are TFs, including *Hand2* (Fig. 8, green arrows). In particular, *Tbx3* is required to upregulate 5′ Hoxd genes, *Hand2* and *Ets2*, which function to activate and/or upregulate *Shh* expression (this study; Kmita et al., 2005; Galli et al., 2010; Lettice et al., 2012). As TBX3 acts predominantly as a repressor (Carlson et al., 2001), positive regulation must depend on interactions with other transcriptional regulators. Indeed, a recent study established that TBX3 interacts with BCL9 (as part of WNT/β-catenin transcriptional complexes), which upregulates gene expression during forelimb bud outgrowth (E10.5; Zimmerli et al., 2020). Furthermore, WNT signalling has been implicated in formation of chicken limb buds (Kawakami et al., 2001), but to date there is no genetic evidence for an equivalent role in early mouse limb buds. It is also possible that TBX3 interacts with other TBX TFs as during mouse kidney development, whereby TBX2 and TBX3 function downstream of canonical WNT signal transduction in the ureteric mesenchyme (Aydoğdu et al., 2018). This is relevant to limb bud development, as *Tbx2* and *Tbx3* are co-expressed in the anterior and posterior mesenchyme and *Tbx2* expression is not altered in *Tbx3* mutant limb buds (this study). Indeed, stepwise reduction of the *Tbx2* and *Tbx3* gene dosage increases the severity of the limb skeletal malformations, which are not strictly additive but in parts synergistic (Lopatka and Moon, 2022). As the core T-box motif enriched in the TBX3 cistrome is shared by different TBX proteins, co-regulation of target genes in limb buds by several Tbx genes is likely. For example, *Tbx18*, *Tbx15* and *Tbx5*, which are expressed in a rather complementary fashion to *Tbx2/*3 in early limb buds (Sheeba and Logan, 2017), may also regulate TBX3 target genes. In particular, their expression patterns significantly overlap the spatial activity patterns of the *Gli3* enhancers and *Gli3* transcripts. Finally, *de novo* motif analysis reveals enrichment of other types of TF motifs in the TBX3 cistrome, such HOX and TBX TFs, that extensively co-bind T-box-Hox composite motifs in the mouse genome during limb bud development, which serves to integrate their inputs into target gene regulation (Jain et al., 2018).

Most importantly, this study establishes that *Tbx3* functions downstream of *Hand2* and is the TF required to establish the posterior expression boundaries of anterior-proximal and anterior-distally expressed genes (Fig. 8). In wild-type limb buds, sharp posterior and non-overlapping expression boundaries with *Tbx3* are observed for *Gli3*, *Alx4*, *Hand1*, *Irx3* and *Irx5*, and their expression expands into the posterior flank in *Tbx3*^Δ/Δc^ and *Hand2*^Δ/Δc^ limb buds. Comparative analysis of *Gli3* enhancer activities and *Gli3* expression reveals the precision by which TBX3 controls the posterior expression boundary via the two enhancers that provide *Gli3* expression with robustness (Fig. 8, red lines; Osterwalder et al., 2018). The results of the present study are consistent with previous analysis of other loss-of function mutations that act upstream of *Hand2* and *Tbx3.* Inactivation of all Hox9 paralogues and *Isl1* disrupts *Hand2* expression in fore- and hindlimb buds, respectively, which results in posterior expansion of *Gli3* expression and disrupts *Shh* expression (Xu and Wellik, 2011; Itou et al., 2012; this study). The TALE homeodomain TFs *Meis1* and *Meis2* are required for PD limb bud axis patterning, but genetic analysis reveals an additional early requirement of *Meis1/2* in AP limb bud axis patterning (Delgado et al., 2020, 2021). Both MEIS TFs are required for upregulation of 5′ Hoxd expression and activation of *Hand2* expression in the posterior limb bud mesenchyme. In particular, MEIS proteins interact with an enhancer that is required for *Hand2* expression (Delgado et al., 2021). In forelimb buds lacking both *HoxA* and *HoxD* gene functions, *Hand2* remains expressed whereas *Shh* activation is disrupted (Kmita et al., 2005), which indicates that *HoxA* and *HoxD* genes function downstream of *Hand2* and *Tbx3* (this study). These studies reveal the genetic hierarchies and molecular interactions that converge on activation and upregulation of *Hand2* expression, which is in turn required to activate *Tbx3* expression in the posterior limb bud mesenchyme. As the *Hand2* expression domain is larger than that of *Tbx3*, other transcriptional regulators must contribute to refining the *Tbx3* expression domain in the posterior mesenchyme. The importance of generating these expression boundaries and the posterior mesenchymal territory has been corroborated by several studies showing that initially *Hand2* (via *Tbx3*) and subsequently SHH (by inhibition of GLI3 processing) overcome the ‘ground state’ GLI3R repression in the early limb mesenchyme (te Welscher et al., 2002b; Osterwalder et al., 2014). The establishment of this posterior GLI3R-free territory enables activation of SHH signalling, which patterns the future digits at an early stage and promotes distal limb bud outgrowth (Litingtung et al., 2002; te Welscher et al., 2002b; Zhu et al., 2008, 2022).

The present study unveils a previously unrecognised essential role of TBX3 in shaping the posterior expression boundary of anterior genes, including *Irx3/5*. Interestingly, the *Drosophila Tbx2/3* orthologue *optomotor blind* [*omb* (current symbol *bifid*)] is expressed in a complementary manner to the *Drosophila Irx* orthologue *Iroquois-C* (*Iro-C*) genes (*mirr*, *ara*, *caup*) in the wing imaginal disc, which separates the wing hinge and notum territories (Wang et al., 2016). Loss- and gain-of-function analysis by Wang and co-workers (Wang et al., 2016) showed that *omb* directly represses *Iro-C* genes, which is both necessary and sufficient for formation of the boundary fold between the hinge and notum in the wing imaginal disc. Thus, the repression of *Irx3* and *Irx5* by TBX3 is an evolutionarily ancient gene regulatory circuit required for insect wing and leg imaginal disc development (Pflugfelder et al., 2017) that has been co-opted to the establishment of the AP axis and the posterior mesenchymal territory in vertebrate limb buds.

## MATERIALS AND METHODS

### Ethics statement, mouse strains and embryos

All experiments were conducted with the different *Tbx3* and *Hand2* alleles bred into Swiss albino mice (*Mus musculus*) as only robust phenotypes manifest in this strain background with large litter sizes (15-20 embryos), which is in line with the refine and reduce 3R principles. Embryos of both sexes at the developmental ages indicated were used for experimental analysis in accordance Swiss laws and the 3R principles. Embryos were staged by counting somites during dissection, taking into account that the most posterior somite in the forelimb bud field is somite 13 and somite 30 in the hindlimb bud (Martin, 1990). Before initiating an experiment, somite counts were verified and limb bud sizes matched. All animal studies were evaluated and approved by the Regional Commission on Animal Experimentation and the Cantonal Veterinary Office of Basel (national licence 1951). In addition to the *Tbx3*^ΔVenus^ null allele (Kunasegaran et al., 2014), the *Prrx1-Cre* strain (Logan et al., 2002) was used to conditionally inactivate the floxed *Tbx3* (Frank et al., 2013) and *Hand2* (Galli et al., 2010) alleles in the mouse forelimb bud mesenchyme. Wild-type mice and littermates carrying the *Prrx1-Cre* transgene were used as controls. Primers are listed in Table S10.

### Generation of the *Tbx3*^3xF^ mouse allele

The 3xFLAG epitope tag was inserted in frame into the carboxy-terminal exon of the *Tbx3* open reading frame. CRISPR/Cas9-mediated homology-directed repair in combination with a 200 bp single-stranded DNA oligonucleotide (ssODNs) repair template that also encodes the sequence for the triple FLAG peptide. Mouse G4 embryonic stem cells (ESCs) were transfected with a mix containing the targeting vector that also encodes the Cas9 nuclease and the relevant single guide (sg)RNAs together with ssODN repair template. After recovery from transfection, cells were selected with 2 mg/ml puromycin for 48 h to enrich ESCs that have taken up the DNA mix, including the targeting vector. After picking and expanding 100-150 ESC clones, correct genome-editing events were detected by extensive PCR analysis. Correctly genome-edited ESC clones were further verified by sequencing the genomic region of the *Tbx3* locus carrying the 3xFLAG epitope insertion. Verified ESC clones were used to generate several aggregation chimeras. Highly chimeric mice (≥70%) were then bred to germline and the genome-edited *Tbx3* verified again. Primers are listed in Table S10.

### Skeletal analysis

Embryos were collected at E14.5 into ice-cold PBS and fixed for 3-5 days in 95% ethanol. The ethanol was then removed and the embryos stained for 24 h with filtered Alcian Blue staining solution prepared by dissolving 30 mg of Alcian Blue 8GX (Sigma-Aldrich, A3157) in 80 ml of 95% ethanol and 20 ml of glacial acetic acid. Then, they were rinsed twice for 15 min in 95% ethanol and stored for 24 h in 95% ethanol. The samples were then cleared in 1% KOH w/v in water for 90 min to 3 h. This was followed by counter-staining bone with Alizarin Red (50 mg of Alizarin Red per litre 1% KOH) for 3 h. Clearing was continued in 1% KOH solution for 30 min, followed by changing the solution as follows to v/v ratios of 1% KOH/glycerol: 80/20 for 4 days or until the embryos have cleared, then the solution was changed every 48 h to 60/40, 40/60 and samples were stored in 20/80 1% KOH/glycerol. Images were taken using a Leica MZ-FL2 stereo microscope. For each genotype, at least three biological replicates were analysed.

### Western blot experiments

Protein extracts were prepared from forelimb buds (E10.5, 33-36 somites) and 15 mg of total protein per sample loaded onto 12.5% SDS-PAGE gels. Following gel-electrophoresis, proteins were transferred to a PVDF membrane (Merck, IPVH00010) using a semi-dry transfer cell (Bio-Rad Transblot SD). TBX3^3xFLAG^ proteins were detected by chemo-luminescence using monoclonal M2 anti-FLAG antibodies (1:500; Sigma-Aldrich, F1804, P-code: 1003505871) in combination with donkey anti-mouse IgG-HRP antibodies (1:5000; Millipore, AP192P, lot: 1975925) using a ChemiDocTM XRS+ with Image Lab from Bio-Rad. TBX3 proteins were detected using rabbit anti-TBX3 antibodies (1:500; Invitrogen, 42-4800, lot: WA317227) with secondary goat anti-rabbit HRP antibodies (1:5000; Millipore, AP187P, lot:1988571).

### TBX3^3xF^ ChIP-seq analysis

Two independent biological replicates were analysed separately to ensure reproducibility following ENCODE guidelines. For each replicate, forelimb buds of ∼70 *Tbx3^3xF/3xF^* mouse embryos at E9.75-E10.25 (29-32 somites) were dissected in ice-cold PBS and crosslinked in 1% formaldehyde for 10 min (Sheth et al., 2016). Fixation of limb buds was stopped with glycine, and limb buds were washed in PBS (with protease inhibitors) and stored at −80°C. Frozen tissues were thawed on ice, nuclear extracts prepared and chromatin sheared by sonication to a size range of ∼200-500 base pairs. Per replicate, 5 mg of mouse-M2-anti-FLAG antibodies (Sigma-Aldrich, F1804, P-code: 1003505871) coupled to protein A/G beads were used for chromatin immunoprecipitation. After overnight incubation, the immunoprecipitated chromatin complexes were extensively washed, and the chromatin complexes eluted from the beads and the DNA purified after decrosslinking. After assessing the quality of the eluted DNA, libraries for sequencing were prepared using the MicroPlex library preparation kit v2 (C05010014, Diagenode). Libraries were purified using AMPure XP purification beads (A63880, Beckman Coulter Life Sciences) and sequenced using the Illumina NextSeq 500 system.

### ChIP-seq analysis for identification of enriched regions

Paired-end reads of 75 bp from TBX3 ChIP-seq experiments were subjected to quality checking using FastQC v0.11.4 (https://github.com/s-andrews/FastQC/). Trim_Galore v0.4.1 wrapper tool for Cutadapt (Martin, 2011) was used to remove adaptor contamination. High-quality reads were mapped to mouse mm10 reference genome using Bowtie2 v2.2.9 (Langmead et al., 2009). After alignment, PCR duplicates were removed using Picard v2.9.2 (https://github.com/broadinstitute/picard) and orphan reads were also discarded. Finally, uniquely mapped reads in proper pairs were extracted using SAMtools v1.7 (Li et al., 2009). To perform peak calling on each replicate (*n*=2 biological replicates), MACS2 v2.1.1 (Zhang et al., 2008) was used with settings -g mm -p 1e-3 --nomodel --extsize --call-summits -B --SPMR. The value of --extsize was set based on MACS2 predicted fragment length. To obtain coverage tracks per replicate, the --SPMR option of MACS2 was used to generate a signal file of fragment pileup per million reads. Evidence from biological replicates were combined using MSPC v2 with parameters -r biological -s 1E-5 -W 1E-2. The resultant peaks were assigned the lowest *P*-value of the overlapping peaks across both replicates. Peaks corresponding to X, Y and M chromosomes were discarded and only reproducible peaks from both replicates were used as consensus peak set (Jalili et al., 2018). This resulted in identification of 11,422 regions significantly enriched in TBX3^3xF^ chromatin complexes that were used for further analysis. The raw sequencing data of the HAND2^3XF^ ChIP-seq from mouse limb buds at E10.5 (GSE55707; Osterwalder et al., 2014) were subjected to quality checking using FastQC v0.11.4 (Andrews, 2010) and Trim_Galore v0.4.1 wrapper tool for Cutadapt (Martin, 2011). High-quality reads were mapped to the mouse mm10 genome assembly using Bowtie2 v2.2.9 (Langmead and Salzberg, 2012). After alignment, PCR duplicates and orphan reads were discarded using SAMtools v1.7 (Li et al., 2009) and only uniquely mapped reads retained. Subsequently, MACS2 v2.1.1 (Zhang et al., 2008) was used with settings -g mm -p 1e-3 --nomodel --extsize 200 --call-summits -B –SPMR to perform peak calling. Peaks corresponding to X, Y and M chromosomes were discarded, which yielded 19,639 genomic regions significantly enriched in HAND2^3xF^ chromatin complexes.

### Annotation and evolutionary conservation analysis

The significantly enriched TBX3^3xF^ ChIP-seq peaks were annotated to promoter, intergenic and intragenic regions out using the annotatePeaks.pl utility available as part of HOMER (Heinz et al., 2010) and their evolutionary conservation was determined using the phastCons conservation scores. The genome-wide track (track name: mm10.60way.phastCons60wayPlacental.bw) of the base-pair phastCons scores in placental mammals was downloaded from the UCSC genome browser (Tyner et al., 2017). The base-pair scores for the 300 bp flanking regions on either side of the TBX3^3xF^ peaks was determined using bwtool (Pohl and Beato, 2014).

### Motif enrichment analysis

To scan for consensus TF binding sites and *de novo* motifs in the 3057 TBX3 ChIP-seq peaks that are located in open chromatin regions (i.e. overlapping ATAC-seq peaks; Table S2), the *findMotifsGenome.pl* utility of the HOMER suite (v4.11; Heinz et al., 2010) was used with the following parameters: the size of the region used to identify motifs was defined as number of bases downstream and upstream of the centre of the called ChIP-seq peak: −150,150. The motif length, *len*, was defined as the range from six to 18 bases. HOMER randomly selects background sequences from a pool of gene-proximal regions (±50 kb) for ChIP-Seq analysis and automatically discards genomic regions overlapping with the target peaks. These parameters were used to scan all TBX3 ChIP-seq peaks identified.

### Differential gene expression analysis using RNA-seq

polyA^+^RNAs were prepared from pairs of forelimb buds isolated from wild-type and *Tbx3*-deficient mouse embryos at E9.75-10.0 (28-31 somites). Similarly, polyA^+^ RNAs were extracted from pairs of forelimb buds of wild-type and *Hand2*-deficient limb buds at E10.0-10.25 (31-33 somites). For each genotype, three independent biological replicates were analysed by RNA-seq using the Illumina NextSeq 500 to generate single-end reads of 75 bp length. Raw sequencing reads were subjected to quality checking using FastQC v0.11.4 and high-quality reads were aligned to the mouse (mm10) genome using STAR v2.5.2 (Dobin et al., 2013) aligner with --*twopassMode Basic* and --*quantMode TranscriptomeSAM* settings. Subsequently, the *rsem-calculate-expression* utility of RSEM v1.3.0 (Li and Dewey, 2011) was used to quantify gene expression and transcript levels across all samples. The mouse reference gene annotation in GTF format was obtained from ENSEMBL (release 91). Prior to identification of DEGs, small non-coding RNAs (snoRNA, miRNA, miscRNA, scRNA and scaRNA) were filtered out. To further reduce noise and increase robustness, only genes with counts per million reads mapped (CPM) ≥1 per replicate were maintained. edgeR (Robinson et al., 2010) was used to identify DEGs in wild-type and *Tbx3-* and *Hand2*-deficient forelimb buds. Sequencing libraries were normalised using TMM normalisation and differential expression between pairs of conditions were evaluated using excatTest utility of edgeR. False discovery rates were estimated using the Benjamini–Hochberg correction (Benjamini and Hochberg, 1995). DEGs with an absolute fold change (FC) cutoff of >1.2 and an adjusted *P*-value ≤0.05 were considered as significantly different and included in downstream analysis. Functional enrichment of the biological processes for DEGs was assessed by GO analysis (https://geneontology.org/).

### Identification of TBX3 and HAND2 target genes

The TBX3^3xF^ (*n*=3057) and HAND2^3xF^ (*n*=19,639) ChIP-seq peaks located in regions of accessible/open chromatin, as determined by ATAC-seq analysis (Jhanwar et al., 2021), were associated to their candidate target genes by GREAT analysis (Genomic Regions Enrichment of Annotations Tool; McLean et al., 2010). GREAT analysis was conducted using the mouse mm10 reference genome with advanced options and the rule that genes are located within ≤1 MB of a ChIP-seq peak in accessible chromatin. Among these genes, the ones expressed differentially (DEGs) and associated with TBX3^3xF^ and/or HAND2^3xF^ ChIP-seq peaks were defined as candidate TBX3, HAND2 or common target genes in mouse limb buds.

### GO analysis of DEGs and putative gene targets of TBX3 and HAND2

GO analysis (release 2021-08-18; Gene Ontology Consortium, 2021; Mi et al., 2019) was carried out for the following datasets: (1) up- and downregulated DEGs identified by pairwise comparison of wild-type versus *Tbx3*-deficient and wild-type versus *Hand2*-deficient limb bud samples; and (2) candidate TBX3 and HAND2 target genes. For each set, the biological processes with adjusted *P*-values ≤0.05 were considered as significantly enriched and the top 20 processes are shown in Figs S2 and S4.

### Hierarchical clustering, plots and statistical testing

Clustering, plots and statistics were handled in the statistical computing environment R v3. GO enrichment analysis plots with the top 20 enriched biological processes (FDR≤0.05) were generated using GO analysis (https://geneontology.org/). Heatmaps of DEGs and differentially expressed target genes were prepared using Python v3.7.

### Identification of candidate CRMs enriched in HAND2 and/or TBX3 chromatin complexes associated with shared target genes

To identify the CRMs interacting with either or both transcriptional regulator for all shared target genes, the following datasets were parsed, wrangled and filtered: (1) CRMs enriched in TBX3 chromatin complexes that overlap accessible chromatin and associate with all shared target genes using GREAT for linear proximity association; and (2) by the same approach, association of HAND2-interacting CRMs with all shared target genes. Initially, target genes associated with CRMs that are enriched in both HAND2 and TBX3 chromatin complexes were identified. This identified ten of the 33 shared target genes, of which two, namely *Ets2* and *Hs3st3a1*, were each linked to two CRMs interacting with both HAND2 and TBX3. Next, the following values were computed for each target gene: the number of CRMs enriched either in HAND2 or TBX3 chromatin complexes plus the number of CRMs interacting with both HAND2 and TBX3. The results of this analysis for each shared target gene are visualised in the heatmap shown in Fig. S6.

### Immunofluorescence analysis

#### Frozen sections

Embryos were collected in ice-cold PBS and fixed for 2 h at 4°C in 4% paraformaldehyde (PFA) in PBS. After washing with PBS, samples were then cryoprotected using a gradient of sucrose: 10% sucrose/PBS (w/v), 20% sucrose/PBS, 30% sucrose/PBS (1 h each) at 4°C. Embryos were then embedded in 50:50 (v/v) OCT/30% sucrose. For immunofluorescent staining, 10 µm sections were prepared. *Tbx3*^3xF/3xF^ or wild-type sections were subjected to three 5 min washes in PBS, one 30 min wash with 0.1% (v/v) Tween 20 (Sigma-Aldrich, 93773) in PBS (PBT) and one further 5 min wash in PBS. They were blocked in 1% bovine serum albumin (BSA) in PBT for 1 h at room temperature (RT) and incubated overnight at 4°C with a monoclonal mouse anti-FLAG M2 antibody (Sigma-Alrich, F1804, P-code: 1003505871) diluted 1:500 in 1% BSA/PBS. Sections were subjected to three 5 min washes in PBS, followed by one in PBT and were then incubated in the dark for 60 min at RT with an Alexa Fluor 488-conjugated goat anti-mouse secondary antibody (Invitrogen, A-11007) diluted 1:500 in 1% BSA/PBS. Sections were finally subjected to three 10 min washes in PBS, and one in PBT (5 min), then nuclei were counterstained in 1 µg/ml Hoechst 33258/PBS (5 min) followed by a further three 5 min washes in PBS. They were then mounted in Mowiol 4-88 and dried overnight in the dark.

#### Whole-mount immunofluorescence analysis

After fixation in 4% PFA/PBS for 24 h, embryos were kept in storage buffer (PBS) with 0.005% sodium azide. To start the experiment, embryos were washed with PBS (three 5 min washes) and permeabilised in 0.5% Triton X-100 in PBS for 60 min with shaking at RT. Then, the samples were blocked in 1% BSA, 0.1%Tween-20 with 5% serum in 0.5% Triton X-100 in PBS for 45 min. The samples were then incubated with primary rabbit polyclonal anti-TBX3 antibodies (1:200; Abcam, ab99302, lot: GR3354374-1) in blocking buffer for 3 days at 4°C with gentle shaking. After six washes in 0.5% Triton X-100 in PBS, the samples were incubated with Alexa Fluor 647-conjugated donkey anti-rabbit secondary antibodies (1:1000; Life Science Technologies, A31573, lot: 2674379) in 1% BSA, 5% serum and 0.5% Triton X-100 in PBS for 2 days at 4°C with gentle shaking. After six washes in 0.5% Triton X-100 in PBS, the samples were incubated and then the embryos were counterstained with DAPI (1:1000; Sigma-Aldrich, D9542) in 0.5% Triton X-100 in PBS overnight at 4°C. Finally, the samples were cleared in 2.5 M fructose-glycerol 60% (v/v) (fructose, Sigma-Aldrich, F0127; glycerol, AppliChem, 131339) and mounted onto microscope slides (ThermoFisher Scientific, J1800AMNZ) and secured with coverslips (VWR, 631-0153) (Morabito et al., 2023). Images were acquired as described below.

### WISH

For each genotype and gene, at least *n*=3 independent biological samples were analysed. Embryos were collected in cold PBS and then fixed for 24 h in 4% PFA. Then, they were gradually dehydrated into 100% methanol and stored at −20°C. To start the experiment, a pool of marked wild-type and mutant embryos with matched limb buds were placed in one tube per probe to allow for direct comparison of results. Next, the embryos were rehydrated and washed twice in PBT. After bleaching in 6% H_2_O_2_ in PBT for 15 min at RT, they were washed three times in PBT (5 min each) and treated with proteinase K (10 µg/ml) for 10 (E10.0) or 15 (E10.5) min. The proteinase K digestion is inactivated in 2 mg/ml glycine in PBS for 5 min at RT. Then, embryos were washed twice with PBT for 5 min each wash and then re-fixed in 4% PFA/0.2% glutaraldehyde in PBT for 20 min. After two washes in PBT at RT, the embryos were pre-blocked to reduce background in prehybridisation buffer for at least 60 min at 70°C. For each gene of interest, 10 µl of riboprobe was diluted in 1 ml of prehybridisation buffer per tube, denatured at 85°C for 5 min, quenched on ice and added to the samples after the removing the prehybridisation solution and hybridising overnight at 70°C.

The next day, samples were washed in 800 µl prehybridisation buffer for 5 min at 70°C, then each 5 min 400 µl of 2×SSC buffer was added (three times) at 70°C. This was followed by two washes (30 min each) in 2×SSC, 0.1% CHAPS at 70°C. An RNase treatment was carried out for 45 min in 2×SCC, 0.1% CHAPS and 20 µl/ml RNase A. Then, samples were washed twice in maleic acid (pH 5.2) for 10 min at RT, then twice for 30 min each wash with maleic acid at 70°C. This was followed by three 5 min washes with 0.1% Tween 20 in TBS (TBST) and pre-blocking in 10% w/v lamb serum in TBST for 1 h at RT. The blocking solution was exchanged with anti-digoxigenin-AP Fab fragments (Sigma-Aldrich, 11093274910) diluted 1:2000 in 1% w/v lamb serum in TBST and incubated overnight at 4°C.

After extensive washes in TBST (three 5 min washes and then eight 30 min washes), the samples were equilibrated for three 10 min incubations in NTMT buffer (2 ml 5 M NaCl, 5 ml Tris-HCl pH 9.5, 5 ml 1 M MgCl_2_, 1 ml Tween 20 in 100 ml H_2_O). The signal was developed in BM Purple (Roche, 11442074001) in the dark and the signal checked regularly until the best possible signal-to-noise ratio was achieved. Weak signals can be developed overnight at 4°C. The development was stopped by five 5 min washes in PBT and then the PBT was replaced by PBS. For long-term storage, samples were stored in 2% PFA in PBS at 4°C. Images were acquired using a Nikon SMZ25 stereo microscope with DS-Ri2 camera and the NIS-Elements BR5.11.00 software or a Leica MZ FL2 stereomicroscope with the Leica Application Suite X software.

### HCR™ RNA-FISH

The mouse HCR™ probe sets for the different mouse genes analysed and *lacZ* mRNAs together with amplifiers and buffers were purchased from Molecular Instruments. Briefly, embryos were fixed in freshly prepared 4% PFA overnight at 4°C and dehydrated into 100% methanol for storage at −20°C. The HCR™ analysis was performed as described in a recently published step-by step protocol (Morabito et al., 2023) with the following small modifications: the photochemical bleaching was carried out for only 1 h and the clearing before embedding was performed overnight using refractive index matching solution (RIMS) with an RI of 1.45 for confocal imaging.

### Fluorescent image analysis

Immunofluorescent images of sections were acquired using the Leica SP5 confocal microscope and software, and processed using Fiji software. Whole limb buds (immunofluorescence and HCR™) were imaged using a confocal spinning disc (Nikon Ti-E, Hamamtsu Flash 4.0 v2 CMOS camera). The image acquisition software VisiView Premier was used. For different conditions of an experiment, images were acquired with the same settings as they were all in the same tube. To compare pattern changes, the pixel range (min-max) for a given gene was the same across all genotypes within an experiment and the same embryonic stage. Background or low signal was removed for pixel values ≤250 for all genes analysed, with exception of *Gli3* for which the threshold was ≤500 pixels. When all three signals had to be displayed, we used the classic red/green/blue combination, which is the only one that allows the different colour combinations when working with three different overlapping signals.

### Mutations of the TBX3 and HAND2 motifs in the *Gli3* mouse core enhancers *mm1179* and *mm-hs1586* and generation of mouse *lacZ* founder embryos

The TBX3 (this study), HAND2 (Osterwalder et al., 2014), HOXD13 (Sheth et al., 2016), SMAD4 (Gamart et al., 2021) and β-catenin ChIP-seq profiles (R.S., R.Z. and A.Z., unpublished) were used together with the H3K27ac profile to map the core regions in both enhancers. The candidate HAND2-binding sites were mapped using the HAND1::TCF3 motif (Jaspar ID MA0092.1) and the TBX3-binding sites using the human TBX3 motif (Jaspar ID MA1566.1) with ap-value cut-off of 0.01. Two of the most conserved bases of each motif were mutated (Fig. S8C), and the mutated binding regions re-scanned to verify their inactivation as HAND- and TBX-binding regions, respectively. The mutated core enhancer regions (Fig. S9) were then synthesised (Integrated DNA Technologies), inserted into the *Hsp68*-*lacZ* vector (Pennacchio et al., 2006) and founder embryos generated by pronuclear linear DNA injection.

### Generation of mouse *lacZ* reporter strains for two *Gli3* enhancers

The enhancer constructs were generated using a PCR-based strategy from mouse genomic DNA. Primers were designed using Primer3 software. The amplified DNA was inserted into the *Hsp68-lacZ* vector using the Gibson Assembly kit (E2611S, New England Biolabs). The resulting constructs were verified, injection-grade DNA produced and linearised. The linearised plasmids were used for pronuclear injection by the University of Basel Centre for Transgenic Models (CTM) and all founder mice genotyped using specific primers. Two founder mice were obtained for the *Gli3 mm1179* and three for the *mm-hs1586* enhancer. Initial analysis to verify their known enhancer activities was carried out before crossing one line each into our *Tbx3^flox^* line to comparatively analyse the enhancer activities in wild-type and in *Tbx3* mutant mouse embryos. All primers are listed in Table S10.

### Analysis of transgenic *lacZ* embryos

It is standard to determine tissue-specific enhancer activity in at least three independent transgenic embryos expressing the *lacZ* reporter in the tissue of interest (Visel et al., 2007). Embryos were collected in ice-cold PBS and then fixed in 1% formaldehyde, 0.2% glutaraldehyde, 0.2% NP40, 0.01 sodium deoxycholate in PBS for 20 min at 4°C. After three washes in PBS at RT, the embryos were stained at 37°C in the dark with gentle rotation. The staining solution consisted of 0.5 mg/ml 5-bromo-4-chloro-3-indolyl β-D-galactopyranoside (X-Gal) in dimethyl formamide, 0.25 mM K_3_Fe(CN_6_), 0.25 mM K_4_Fe(CN_6_), 0.01% NP40 and 4 mM magnesium chloride. Embryos were checked for the blue staining caused by β-galactosidase activity after about 90 min and then every 40-60 min thereafter. If the staining was weak or absent, the staining solution was replaced and the development was continued overnight at 37°C in the dark. The next day, embryos were washed in PBS and post-fixed in 4% PFA/0.1% glutaraldehyde in PBS for 1 h, followed by two washes in PBS, and then stored in PBS with 0.05% sodium azide at 4°C. Samples were analysed and *z*-stack images acquired using a Nikon SMZ25 stereomicroscope equipped with a DS-Ri2 camera and NIS-Elements BR5.11.00 software.

### Available datasets used in this study

A list of genes expressed in mouse limb buds and/or for which mutagenesis, loss of function and gain of function causes congenital limb malformations was manually curated using based on information available in the *embrys* and the Mouse Genome Informatics (MGI) databases. The raw sequencing ATAC-seq data from mouse forelimb buds at E9.75 corresponds to the dataset published by Jhanwar et al. (2021) (GSE164736). The HAND2 ChIP-seq dataset from mouse limb buds at E10.5 was from Osterwalder et al. (2014) (GSE55707). The histone modification ChIP-seq datasets (H3K27me3, GSE86767 and H3K27ac, GSE86760) for mouse limb buds at E10.5 are from the mouse ENCODE project.

## Supplementary Material



10.1242/develop.202722_sup1 Supplementary information

Table S1. Annotation of curated TBX3 ChIP-seq peaks to nearest promoter and genomic features.

Table S2. Association of the curated TBX3 ChIP-seq peaks to the two nearest genes (range ≤1Mb).

Table S3. List of differentially expressed genes between wildtype and T*bx3*^Δ/Δ^ limb buds at E9.75-10.0.

Table S4. List of differentially expressed TBX3 target genes.

Table S5. Manually curated functional annotation of TBX3 target genes. Grey shaded genes are expressed during limb bud development. Genes indicated in bold are transcription factors.

Table S6. TBX3 target genes that are also differentially expressed in wildtype versus *Shh*-deficient limb buds at E10.5.

Table S7. List of differentially expressed genes between wildtype and *Hand2*^Δ/Δ^ limb buds at E9.75-10.0.

Table S8. List of differentially expressed HAND2 target genes.

Table S9. Shared TBX3 and HAND2 target genes.

Table S10. Primers for genotyping mouse strains.
