# Supplementary material for: TBX3 is essential for establishment of the posterior boundary of anterior genes and upregulation of posterior genes together with HAND2 during the onset of limb bud development
Source: Development. 2024 Jun 3;151(11):dev202722. doi: 10.1242/dev.202722 (PMC11190573; doi:10.1242/dev.202722)
Supplement: Supplementary file 1 [file develop-151-202722_review_history.pdf]

TBX3 is essential to establish the posterior boundary of anterior genes and up-regulate posterior genes with HAND2 during onset of limb bud development

Geoffrey Soussi, Ausra Girdziusaite, Shalu Jhanwar, Victorio Palacio, Marco Notaro, Rushikesh Sheth, Rolf Zeller and Aimee Zuniga

DOI: 10.1242/dev.202722

Editor: Liz Robertson

Review timeline

Original submission:	21 January 2024
Editorial decision:	27 February 2024
First revision received:	3 April 2024
Accepted:	26 April 2024

Original submission

First decision letter

MS ID#: DEVELOP/2024/202722

MS TITLE: TBX3 is essential to establish the posterior boundary of anterior genes and up-regulate posterior genes with HAND2 during onset of limb bud development

AUTHORS: Geoffrey Soussi, Ausra Girdziusaite, Shalu Jhanwar, Victorio Palacio, Rushikesh Sheth, Rolf Zeller, and Aimee Zuniga

I have now received all the referees reports on the above manuscript, and have reached a decision. The referees' comments are appended below, or you can access them online: please go to BenchPress and click on the 'Manuscripts with Decisions' queue in the Author Area.

The overall evaluation is positive and we would like to publish a revised manuscript in Development, provided that the referees' comments can be satisfactorily addressed. Please attend to all of the reviewers' comments in your revised manuscript and detail them in your point-by-point response. If you do not agree with any of their criticisms or suggestions explain clearly why this is so. If it would be helpful, you are welcome to contact us to discuss your revision in greater detail. Please send us a point-by-point response indicating your plans for addressing the referees' comments, and we will look over this and provide further guidance.

Reviewer 1

Advance summary and potential significance to field

In this study, the authors delve into the role of Tbx3 in governing AP patterning during the early stages of limb bud development. With this aim they generate a mouse Tbx3 tagged allele, employing it for ChIP-seq analysis. By integrating this approach with ATAC-seq and transcriptomic analysis in both wild-type and Tbx3 mutants, the authors pinpoint a collection of genes directly targeted by Tbx3. They subsequently juxtapose Tbx3 target genes with those of Hand2 to identify any overlapping candidates. Through spatial analysis of target gene expression and enhancer

© 2024. Published by The Company of Biologists under the terms of the Creative Commons Attribution License (https://creativecommons.org/licenses/by/4.0/).

activity, the authors conclude that Tbx3 plays a pivotal role in setting the posterior boundary of anterior genes impeding their extension into the posterior mesoderm.

This study represents a significant contribution to our comprehension of Tbx3's role in limb development and the establishment of the AP axis highlighting its role in orchestrating precise patterning. The HCR are beautiful and the videos very demonstrative of the boundary between gene expression domains. However, I would like to direct the authors' attention to several points that in my opinion need improvement prior to publication.

Comments for the author

In this study, the authors delve into the role of Tbx3 in governing AP patterning during the early stages of limb bud development. With this aim they generate a mouse Tbx3 tagged allele, employing it for ChIP-seq analysis. By integrating this approach with ATAC-seq and transcriptomic analysis in both wild-type and Tbx3 mutants, the authors pinpoint a collection of genes directly targeted by Tbx3. They subsequently juxtapose Tbx3 target genes with those of Hand2 to identify any overlapping candidates. Through spatial analysis of target gene expression and enhancer activity, the authors conclude that Tbx3 plays a pivotal role in setting the posterior boundary of anterior genes impeding their extension into the posterior mesoderm.

This study represents a significant contribution to our comprehension of Tbx3's role in limb development and the establishment of the AP axis highlighting its role in orchestrating precise patterning. The HCR are beautiful and the videos very demonstrative of the boundary between gene expression domains. However, I would like to direct the authors' attention to several points that in my opinion need improvement prior to publication.

Major points:

- The authors mention that recent results (Lex et al., 2022) have “cast doubt on the postulated repressive function of the predominant GLI3R isoform upstream on Hand2 prior to activation of SHH signaling”. Given the significant implication of Gli3R function as a repressor prior to Shh activation and the and the assertive conclusions drawn in Lex et al., 2022, a thorough discussion of this aspect is warranted.
- Figure 3 illustrates a spatial analysis of Tbx3 direct target genes categorized as either upregulated or downregulated by Tbx3. While for some genes, such as Hand2, Prdm1, and Hoxd13, it is evident that expression decreases in the absence of Tbx3, others present more subtle alterations. For example, Sall3 appears uniformly downregulated and the Lmo1 expression domain seems not to overlap with that of Tbx3.

Additionally, for several genes repressed by Tbx3, such as Tbx2, Cddh3, and Aldh1a2, the alterations in expression are really subtle.

- Does the analysis in Figure 3C encompass the function of the anterior domain of Tbx3, or is this section exclusively focused on the posterior domain? I would like some consideration of the anterior Tbx3 domain function, which initiates later and has a more limited extension and lower expression level compared to the posterior domain. Is it possible that Tbx3 regulates a single gene differently in its anterior and posterior domains?
- Aldh1a2 is known to depend on AER-Fgf. The alteration in absence of Tbx3 is significant raising the question on whether the AER may be altered in Tbx3 mutants. Have the authors investigated this possibility?
- What is the intended meaning of this statement in the Abstract "Here, we identify TBX3 as the transcription factor to initiate AP axis polarity in mouse"? Are the authors suggesting that Tbx3 acts upstream of Hand2? The cross analysis in the respective mutants doesn’t seem to support this.
- If Tbx3 is never detected in the absence of Hand2, would it be possible to distinguish Tbx3-specific from Tbx3-Hand2 shared genes? it would be feasible to identify genes downstream of Hand2 that are independent of Tbx3.
- Do the Tbx3-bound and Hand2-bound cis-regulatory modules in target genes coincide? In other words do both transcription factors cooperate within a single enhancer, or do they modulate the same gene through independent enhancers?
- Among the common genes is Pitx1, a known hindlimb-specific transcription factor. How do the authors explain this finding?

© 2024. Published by The Company of Biologists under the terms of the Creative Commons Attribution License (https://creativecommons.org/licenses/by/4.0/).

- I would suggest the authors consider adding a concluding schematic diagram illustrating the new connections they have uncovered in this study.

Minor points:
- If I understood correctly, the authors have employed a 100 Kb threshold to differentiate between peaks classified as close or distal. However, it's worth noting that peaks located much closer than 100 Kb to the TSS are typically regarded as distal.
- Figure S1 Western blot: Does "WE" refer to whole embryo? Shouldn't the AntiTBX3 label be positioned in the upper part, similar to anti-flag?
- Starting at the bottom of page 9, the authors manually select a subset of target genes based on the literature, which they refer to as "annotated TBX3 genes." I find this terminology a bit confusing and suggest considering an alternative term to refer to this subset of targets.
- In Figure 7A, is the mm1179 region (red bar) the region "required" for enhancer activity, or is it simply the region that was tested?
- Regarding the WMISHs in Figure 3 (WT), the Hoxd13 WMISH appears rather poor, and Hand1 seems to lack the anterior bias.

Methods:
- Please detail how do you determine somite number in the embryos, is some reference used?
- Were both replicates of the Tbx3 ChIP-seq datasets analyzed together?
- Please indicate in the text that the Hand2 ChIP-seq peaks were also intersected with the ATAC peaks to remove those regions not opened, as done with Tbx3.

Reviewer 2

Advance summary and potential significance to field

In this manuscript, the authors explain in greater detail how TBX3 regulates anterior-posterior axis formation in the limb bud. They generated reporter mouse strains to perform ChIP-seq and enhancer analyses to convincingly show how TBX3 sets an anteroposterior boundary in the early limb bud. New information is beautifully integrated with earlier data throughout the results and the discussion such that this paper will be essential reading for those interested in axis specification. The discussion regarding potential transcriptional coactivation of patterning targets underscores there is still much to learn about gene regulatory networks and raises new hypotheses for the field. I think the manuscript can be published in its current form.

Comments for the author

For the non-limb specialist, perhaps the introductory logic regarding why additional transcriptional regulators that set the Gli3 and Irx3/5 boundaries need to be invoked can be clearer.

The axial/conceptual similarity between human versus mouse deficiency of TBX/Tbx leading to posterior deficiency or anterior expansion can also be clarified.

At the outset, it might be useful to state explicitly that Tbx3 is expressed within the posterior Hand2 domain (in addition to being a target of HAND2).

It may be useful to specify in the text that TBX3 coregulation of hedgehog targets are predominantly GliA or GliR dependent.

First revision

Author response to reviewers' comments

Reviewer 1 Advance Summary and Potential Significance to Field:
In this study, the authors delve into the role of Tbx3 in governing AP patterning during the early

© 2024. Published by The Company of Biologists under the terms of the Creative Commons Attribution License (https://creativecommons.org/licenses/by/4.0/).

stages of limb bud development. With this aim they generate a mouse Tbx3 tagged allele, employing it for ChIP-seq analysis. By integrating this approach with ATAC-seq and transcriptomic analysis in both wild-type and Tbx3 mutants, the authors pinpoint a collection of genes directly targeted by Tbx3. They subsequently juxtapose Tbx3 target genes with those of Hand2 to identify any overlapping candidates. Through spatial analysis of target gene expression and enhancer activity, the authors conclude that Tbx3 plays a pivotal role in setting the posterior boundary of anterior genes impeding their extension into the posterior mesoderm.

This study represents a significant contribution to our comprehension of Tbx3's role in limb development and the establishment of the AP axis highlighting its role in orchestrating precise patterning. The HCR are beautiful and the videos very demonstrative of the boundary between gene expression domains. However, I would like to direct the authors' attention to several points that in my opinion need improvement prior to publication.
A: We would like to thank the reviewer for their very positive and insightful evaluation of our study.

Reviewer 1 Comments for the Author:
In this study, the authors delve into the role of Tbx3 in governing AP patterning during the early stages of limb bud development. With this aim they generate a mouse Tbx3 tagged allele, employing it for ChIP-seq analysis. By integrating this approach with ATAC-seq and transcriptomic analysis in both wild-type and Tbx3 mutants, the authors pinpoint a collection of genes directly targeted by Tbx3. They subsequently juxtapose Tbx3 target genes with those of Hand2 to identify any overlapping candidates. Through spatial analysis of target gene expression and enhancer activity, the authors conclude that Tbx3 plays a pivotal role in setting the posterior boundary of anterior genes impeding their extension into the posterior mesoderm.

This study represents a significant contribution to our comprehension of Tbx3's role in limb development and the establishment of the AP axis highlighting its role in orchestrating precise patterning. The HCR are beautiful and the videos very demonstrative of the boundary between gene expression domains. However, I would like to direct the authors' attention to several points that in my opinion need improvement prior to publication.
A: We have addressed all the very valid points and issues raised by this reviewer in revising the manuscript.

Major points:
-The authors mention that recent results (Lex et al., 2022) have "cast doubt on the postulated repressive function of the predominant GLI3R isoform upstream on Hand2 prior to activation of SHH signaling". Given the significant implication of Gli3R function as a repressor prior to Shh activation and the and the assertive conclusions drawn in Lex et al., 2022, a thorough discussion of this aspect is warranted.
A: We have extended our discussion of the study by Lex et al. (page3/4): A study by Lex et al. (2022) showed that the binding of GLI3R to its target CRMs is inert prior to activation of SHH signaling. In *Gli3*-deficient limb buds at early stages, i.e. when GLI3 repression is removed, enhancer accessibilities, activities and target gene expression are not increased as it the case after the onset of SHH signaling (Lex et al. 2022). These findings contrast with the fact that the mutually antagonistic restriction of *Hand2* and *Gli3* does not require SHH signaling, as both the posterior *Hand2* and anterior *Gli3* restriction occur normally in *Shh*-deficient limb buds (Welscher et al.,2002a).

-Figure 3 illustrates a spatial analysis of Tbx3 direct target genes categorized as either upregulated or downregulated by Tbx3. While for some genes, such as Hand2, Prdm1, and Hoxd13, it is evident that expression decreases in the absence of Tbx3, others present more subtle alterations. For example, Sall3 appears uniformly downregulated and the Lmo1 expression domain seems not to overlap with that of Tbx3. Additionally, for several genes repressed by Tbx3, such as Tbx2, Cddh3, and Aldh1a2, the alterations in expression are really subtle.
A: we have modified the text for clarification (page11): The expression domains of all these target genes overlap at least partially with the posterior *Tbx3* domain (Fig. 1C), but it is likely

© 2024. Published by The Company of Biologists under the terms of the Creative Commons Attribution License (https://creativecommons.org/licenses/by/4.0/).

that downstream effects as part of the TBX3-target GRN contribute to the overall reduction of *Sall3* and *Lmo1* expression in the distal mesenchyme (Fig.3 C,D).
Concerning detection of upregulation of gene expression by WISH (page 11): Next, we analysed the spatial expression of target genes up-regulated in *Tbx3*-deficient forelimb buds (Fig. 3B,E). Although detection of transcriptional up-regulation by WISH is more challenging than down-regulation (Gamart et al., 2021), spatial changes are observed for several target genes in *Tbx3*$^{D/Dc}$ forelimb buds (Fig. 3B,E).

-Does the analysis in Figure 3C encompass the function of the anterior domain of Tbx3, or is this section exclusively focused on the posterior domain? I would like some consideration of the anterior Tbx3 domain function, which initiates later and has a more limited extension and lower expression level compared to the posterior domain. Is it possible that Tbx3 regulates a single gene differently in its anterior and posterior domains?
A: this is difficult if not impossible to determine for all target genes. But we have amended the text as follows (page 10): At these stages, *Tbx3* is expressed predominantly in the posterior mesenchyme, with little to no expression in the anterior mesenchyme (E9.75-E10.25, Emechebe et al., 2016 and Fig. 6A). The GRN analysis shows that the majority of target genes expressed in the anterior and proximal mesenchyme and AER are repressed by TBX3. This is also the case for several genes expressed in the distal mesenchyme, while most TBX3 target genes expressed in the posterior mesenchyme are positively regulated. Together this analysis indicates that posterior TBX3 largely governs the target GRN in early limb buds, although a potential contribution from anteriorly expressed TBX3 cannot be ruled out.

-Aldh1a2 is known to depend on AER-Fgf. The alteration in absence of Tbx3 is significant raising the question on whether the AER may be altered in Tbx3 mutants. Have the authors investigated this possibility?
A: we have analyzed AER-Fgf8 and AER-Fgf4 expression in wild-type and mutant limb buds lacking Tbx3 in the mesenchyme (new Fig. S5B) and described the results on page 12 (bottom): As previous analysis showed that AER-FGFs upregulate *Cyp26b1* expression (Probst et al., 2011), we assessed AER-*Fgf8* and *Fgf4* expression in wildtype and *Tbx3*$^{D/Dc}$ forelimb buds, but no changes were detected (Fig. S4B).

-What is the intended meaning of this statement in the Abstract "Here, we identify TBX3 as the transcription factor to initiate AP axis polarity in mouse"? Are the authors suggesting that Tbx3 acts upstream of Hand2? The cross analysis in the respective mutants doesn’t seem to support this.
A: we agree and have changed this sentence in the abstract as follows: Here, we show that TBX3 is required to establish the posterior expression boundary of anterior genes in mouse limb buds.

-If Tbx3 is never detected in the absence of Hand2, would it be possible to distinguish Tbx3-specific from Tbx3-Hand2 shared genes? it would be feasible to identify genes downstream of Hand2 that are independent of Tbx3.
A: in Hand2 mutants only posterior Tbx3 is missing and we show that the posterior restriction of anterior genes depends on posterior Tbx3. Conversely, we provide genetic evidence that the (initial) up-regulation of posterior shared target genes depends on Hand2, and Tbx3 contributes to maintenance. Last but not least due to feedback regulation between Hand2 and Tbx3, i.e. Hand2 expression is reduced in Tbx3 deficient limb buds and vice versa, it is very difficult not to say impossible to answer the above questions.

-Do the Tbx3-bound and Hand2-bound cis-regulatory modules in target genes coincide? In other words, do both transcription factors cooperate within a single enhancer, or do they modulate the same gene through independent enhancers?
A: we have performed this analysis and the results are included in a new Fig. S6. The results are described on page 16: In addition, the interactions of TBX3 and HAND2 with CRMs associated with the shared target genes were mapped (Fig. S6). This established that most target genes (~70%) are regulated by CRMs that either interact with HAND2 or TBX3. Only ~30% of all target gene loci include at least one CRM that is enriched in both HAND2 and TBX3 chromatin complexes (Fig. S6). Taken together, this analysis shows that *Hand2* and *Tbx3* co- regulate major TFs in the GRN that orchestrates limb axes patterning and restricts *Shh* expression to the posterior mesenchyme.

-Among the common genes is Pitx1, a known hindlimb-specific transcription factor. How do the

© 2024. Published by The Company of Biologists under the terms of the Creative Commons Attribution License (https://creativecommons.org/licenses/by/4.0/).

authors explain this finding?
A: the increase in Pitx1 expression levels are very low but significant as it was added to the list by unbiased analysis, however we have no explanation for this. What was likely misleading that is was indicated in bold like other forelimb TFs with spatially restricted expression in forelimb buds- which is obviously wrong. We have now "unbolded" Pitx1 in Fig. 4F and removed it from the text on page 16 (first lines.)

-I would suggest the authors consider adding a concluding schematic diagram illustrating the new connections they have uncovered in this study.
A: That is a great suggestion, we have included a simple scheme highlighting the major novel interactions discovered by this study as new Fig. 8 for the discussion.

Minor points:
- If I understood correctly, the authors have employed a 100 Kb threshold to differentiate between peaks classified as close or distal. However, it's worth noting that peaks located much closer than 100 Kb to the TSS are typically regarded as distal.
A: the description was confusing and has been revised to (page 8): Roughly equal fractions of the $TBX3^{3xF}$ ChIP- seq peaks are located within 0-3kb of the transcriptional start sites (mostly promoter interactions; Zimmerli et al., 2020) and between 3-100kb, which is indicative of intra- and intergenic CRMs with conserved peak summits (Fig. S1D-F and Table S1).

- Figure S1 Western blot: Does "WE" refer to whole embryo? Shouldn't the AntiTBX3 label be positioned in the upper part, similar to anti-flag?
A: corrected in Fig. S1

- Starting at the bottom of page 9, the authors manually select a subset of target genes based on the literature, which they refer to as "annotated TBX3 genes." I find this terminology a bit confusing and suggest considering an alternative term to refer to this subset of targets.
A: corrected to "manually curated" *(now page 10*)

- In Figure 7A, is the mm1179 region (red bar) the region "required" for enhancer activity, or is it simply the region that was tested?
A: the figure legend (page 53) now reads: The enlargements (right panel) show the genomic regions tested for *Gli3* enhancer activity (red bar) and the HAND2 and TBX3 ChIP-seq peaks.

- Regarding the WMISHs in Figure 3 (WT), the Hoxd13 WMISH appears rather poor, and Hand1 seems to lack the anterior bias.
A: agreed- we have repeated the Hoxd13 WMISH and developed longer and replaced the panels. For Hand1 (and Alx4) we also include RNA-FISH analysis in Fig. S4A to better reveal the low Hand1 expression in wildtype forelimb buds at early stages and the posterior expansion of both genes in Tbx3-mutant limb buds.

Methods:
- Please detail how do you determine somite number in the embryos, is some reference used?
A: description on page 24: Embryos were staged by counting somites during dissection taking into account that the most posterior somite in the forelimb bud field is somite 13 and somite 30 in the hindlimb bud (Martin, 1990).

- Were both replicates of the Tbx3 ChIP-seq datasets analyzed together?
A: No - page 26: Two independent biological replicates were analysed separately to ensure reproducibility following ENCODE guidelines.

- Please indicate in the text that the Hand2 ChIP-seq peaks were also intersected with the ATAC peaks to remove those regions not opened, as done with Tbx3.
A: is now stated on page14: Only HAND2 ChIP-seq peaks located in open chromatin regions (ATAC-seq) were analysed in combination with genes expressed differentially in wildtype and *Hand2*$^{\Delta/\Delta c}$ forelimb buds.

Reviewer 2 Advance Summary and Potential Significance to Field:
In this manuscript, the authors explain in greater detail how TBX3 regulates anterior-posterior axis

© 2024. Published by The Company of Biologists under the terms of the Creative Commons Attribution License (https://creativecommons.org/licenses/by/4.0/).

formation in the limb bud. They generated reporter mouse strains to perform ChIP-seq and enhancer analyses to convincingly show how TBX3 sets an anteroposterior boundary in the early limb bud. New information is beautifully integrated with earlier data throughout the results and the discussion such that this paper will be essential reading for those interested in axis specification. The discussion regarding potential transcriptional coactivation of patterning targets underscores there is still much to learn about gene regulatory networks and raises new hypotheses for the field. I think the manuscript can be published in its current form.
A: we would like to thank the reviewer for their appreciation of our study and the comments that we have taken into account for the revisions.

Reviewer 2 Comments for the Author:
For the non-limb specialist, perhaps the introductory logic regarding why additional transcriptional regulators that set the Gli3 and Irx3/5 boundaries need to be invoked can be clearer.
A: we now introduce the need already in the introduction section (page 5): Osterwalder et al. (2014) identified the HAND2 target *cis*-regulatory modules (CRMs) in the genomic landscapes of genes functioning during the onset of limb bud development. Moreover, *Tbx3* was identified as a HAND2 target gene in posterior limb bud mesenchyme, which led to the proposal that in addition HAND2, *Tbx3* might participate in "fine-tuning" the posterior *Gli3* expression boundary (Osterwalder et al., 2014), which prompted identification of the range of TBX3 target genes in early limb buds (this study).

The axial/conceptual similarity between human versus mouse deficiency of TBX/Tbx leading to posterior deficiency or anterior expansion can also be clarified.
A: we expanded the comparative description of UMS and the mouse loss-of-function mutation on page 5/6: Haploinsufficient *TBX3* mutations in humans cause the ulnar-mammary syndrome (UMS), which causes a pleiotropic phenotype including severe reductions of posterior skeletal elements of upper extremities (Bamshad et al., 1999). In contrast to human *TBX3* mutations, inactivation of Tbx3 in the mouse causes mid-gestational lethality, which is circumvented by conditional *Tbx3* inactivation in early mouse limb buds (Davenport et al., 2003; Frank et al., 2013). Similar to the limb skeletal defects observed in human UMS patients, skeletal analysis of *Tbx3*-deficient mouse limb buds shows that the posterior digit 5 and ulna are reduced or lost, but preaxial polydactyly is only detected in mouse mutant limb buds (Bamshad et al., 1999; Emechebe et al., 2016).

At the outset, it might be useful to state explicitly that Tbx3 is expressed within the posterior Hand2 domain (in addition to being a target of HAND2).
A: we have added a sentence on page 6: In the posterior limb bud mesenchyme, *Tbx3* is a HAND2 target gene expressed in a more restricted domain than *Hand2* (Osterwalder et al., 2014)..

It may be useful to specify in the text that TBX3 coregulation of hedgehog targets are predominantly GliA or GliR dependent.
A: this is still not clear as the target range of GLIR and GLIA are to our knowledge unknown in early limb buds (see also our extended discussion of the study by Lex et al. on page 3/4). Others and us have also shown that prior to SHH signaling GLI3 is processed to its repressor from GLI3R.

Second decision letter

MS ID#: DEVELOP/2024/202722

MS TITLE: TBX3 is essential to establish the posterior boundary of anterior genes and up-regulate posterior genes with HAND2 during onset of limb bud development

AUTHORS: Geoffrey Soussi, Ausra Girdziusaite, Shalu Jhanwar, Victorio Palacio, Marco Notaro, Rushikesh Sheth, Rolf Zeller, and Aimee Zuniga

I am happy to tell you that your manuscript has been accepted for publication in Development, pending our standard ethics checks.

© 2024. Published by The Company of Biologists under the terms of the Creative Commons Attribution License (https://creativecommons.org/licenses/by/4.0/).

<u>Reviewer 1</u>

Advance summary and potential significance to field

The authors have effectively addressed all the issues I raised in my previous review, and I believe the paper is now ready for publication (only minor typos remaining).
I would also like to thank the authors for their effort to improving the manuscript.

Comments for the author

The authors have effectively addressed all the issues I raised in my previous review, and I believe the paper is now ready for publication (only minor typos remaining).
I would also like to thank the authors for their effort to improving the manuscript.

<u>Reviewer 2</u>

Advance summary and potential significance to field

>

Comments for the author

The authors have addressed my points and I have no further questions.

© 2024. Published by The Company of Biologists under the terms of the Creative Commons Attribution License (https://creativecommons.org/licenses/by/4.0/).
